# Weighted average algorithm adjusted a novel (1 + FOPI)-FOPI-TID controller structure for AGC with integration of non-linearities and cyber-attack

**DOI:** 10.1038/s41598-026-37004-0

**Published:** 2026-02-02

**Authors:** Moses Awal, Michael Robson Atim, Jimmy Nabende Wanzala, Johnes Obungoloch, Mohamed Barakat

**Affiliations:** 1 Physics Department, Mbarara University of Science and Technology, Mbarara, Uganda; 2 Physics Department, Busitema University, Tororo, Uganda; 3 Biomedical Sciences and Engineering Department, Mbarara University of Science and Technology, Mbarara, Uganda; 4 ECE Department, Giza Engineering Institute, Giza, Egypt; 5 CCE Department, College of Engineering, Almaaqal University, Basrah, Iraq

**Keywords:** Automatic generation control, (1 + FOPI)-FOPI-TID controller, Weighted average algorithm, Cyber-attack, Power systems nonlinearities, Random step load, Energy science and technology, Engineering, Mathematics and computing

## Abstract

The integration of diverse energy sources and the advent of smart grids have intensified the challenges in load frequency management (LFM). Modern power systems are increasingly vulnerable to inherent nonlinearities, such as generation rate constraints, governor dead bands, boiler dynamics, and communication delays, as well as sophisticated cyber-attacks, which collectively threaten frequency stability and tie-line power balance. To address these challenges, this study proposes a novel cascade controller, designated as (1 + FOPI)-FOPI-TID, for robust automatic generation control in hybrid two-area power systems. The controller uniquely combines fractional-order (FO) dynamics with a tilt-integral-derivative stage and is optimized using a green metaheuristic, the weighted average algorithm (WAA). The WAA effectively balances exploration and exploitation to achieve superior parameter tuning. The proposed control architecture processes both area control error (ACE) and frequency deviation (ΔF) signals through dedicated stages, enabling enhanced disturbance rejection and transient response. The system model incorporates a comprehensive set of nonlinearities and evaluates resilience against resonance-based cyber-attacks. Comprehensive simulation studies under both AC and HVDC tie-line configurations demonstrate that the WAA-optimized (1 + FOPI)-FOPI-TID controller significantly outperforms existing schemes, including PD-PI, PIFOD-(1 + PI), and PIDF(1 + FOD). Key performance metrics show a 45.3% reduction in the integral of time-weighted absolute error (ITAE) and improvements in settling times of 47.7% for ΔF₁ and 32.8% for ΔF₂. Sensitivity analysis confirms robustness under ± 25% parameter variations and random load perturbations. During cyber-attacks, the controller maintains the lowest Rate of Change of Frequency (RoCoF), underscoring its dual capability in stabilizing grid dynamics and mitigating cyber-physical threats. These results validate the controller’s potential to enhance operational resilience and reliability in future smart grids.

## Introduction

The stability and efficiency of modern electrical power systems are fundamentally governed by the precise balance between generation and demand, a critical task managed by load frequency control (LFC) or automatic generation control (AGC). As global energy consumption rises and power grids evolve into increasingly interconnected networks, incorporating a diverse mix of conventional thermal plants, hydroelectric facilities, gas turbines, and renewable sources, the challenge of maintaining system frequency within narrow operational limits has become markedly more complex^1,2^. These interconnected systems, while enhancing reliability and enabling economic power exchange, exhibit heightened dynamic sensitivity. Even minor load perturbations can trigger frequency deviations and unscheduled tie-line power flows, potentially leading to instability, equipment stress, and, in extreme cases, cascading failures^3^. Consequently, the development of advanced, robust control strategies for AGC is paramount for ensuring the security and reliability of future power networks.

This imperative is further intensified by two distinct yet converging sets of challenges: inherent physical nonlinearities and emerging cyber-physical vulnerabilities. Traditional linear models often fail to capture the true dynamic behavior of practical systems. Critical physical constraints, including generation rate constraints (GRC) that limit how fast a plant can ramp output, the nonlinear governor dead band (GDB) that causes insensitivity to small speed changes, slow-acting boiler dynamics (BD) in thermal units, and inevitable communication time delays (CTD) in signal transmission, collectively degrade control performance^4–6^. These nonlinearities manifest as increased overshoot, prolonged settling times, and persistent oscillations following disturbances. Simultaneously, the digital transformation of the grid through smart technologies introduces a new frontier of risk. The very communication channels and digital controllers that enable advanced AGC also expose the system to sophisticated cyber-attacks. Threats such as stealthy resonance-based attacks (ResA), which subtly manipulate load signals to drive the system into instability at its natural oscillatory modes, pose a serious and plausible danger^7–9^. This dual challenge, managing complex physical dynamics while fortifying the system against intelligent cyber threats, defines a critical research gap in contemporary AGC design.

In response to the need for improved regulation, control strategies have progressed significantly beyond the ubiquitous Proportional-Integral-Derivative (PID) controller. While PID variants, often tuned via classical or heuristic methods, remain widely used for their simplicity^10^, their performance is frequently inadequate in handling the multifaceted nonlinearities and high variability of modern, multi-source grids^11,12^. This has spurred interest in cascade control (CC) architectures, where two or more controllers are arranged in series, offering superior disturbance rejection and set-point tracking by correcting errors at multiple stages^13^. This evolution highlights the need for more structured and multi-stage control architectures in modern LFM design. The search for optimal parameters for these advanced structures has naturally aligned with the rise of metaheuristic optimization. Algorithms such as TLBO^2^, PSO^14^, Whale optimization algorithm^15^, Jaya optimization^16^, differential evolution^17^, hybrid PSO-BFOA algorithm^18,19^, grey wolf optimization^20^, and others, have been extensively applied to tune AGC controllers, demonstrating the ability to find high-performance solutions in complex, non-convex search spaces.

A parallel and transformative advancement has been the incorporation of Fractional-Order (FO) calculus into control theory. Unlike integer-order controllers, FO controllers possess non-integer differentiation and integration orders, providing additional tuning parameters that grant greater flexibility to shape the dynamic response. This has led to the development of effective strategies like the FOPID controller^21,22^, and more sophisticated cascade designs such as the PIFOD–(1 + PI) and FOPD–FOPI^23,24^ for multi-area systems. The quest for further performance gains continues to yield novel hybrid structures, including tri-stage controllers like (1 + PI)–PI–PID^25^ optimized via enhanced artificial bee colony–particle swarm optimization (IABC–PSO)^25^, hybrid 2DOF–PID–TD controllers^26^, fuzzy-FOPI + PIDN schemes for deregulated two-area systems^27^, and fuzzy-PIDF–(1 + PI) controllers^28^. These developments collectively illustrate the growing shift toward multi-stage and FO-based control architectures for advanced AGC applications.

Despite these considerable advances, a synthesis of the literature reveals several persistent limitations that this work seeks to address. First, many studies validate controller performance under a simplified set of assumptions, often incorporating only one or two nonlinearities. There is a lack of comprehensive evaluation under the simultaneous action of GRC, GDB, BD, and CTD within a hybrid (thermal-hydro-gas-nuclear) generation environment. Second, while cybersecurity is recognized as a paramount concern, very few AGC studies integrate realistic, dynamic cyber-attack models into their simulation-based validation, leaving a gap in understanding controller resilience. Third, the proliferation of complex cascade controller structures is sometimes driven primarily by numerical optimization outcomes, with insufficient explanation of the control-theoretic rationale behind the specific signal flow and component selection. Finally, the claimed superiority of newer metaheuristic optimizers often lacks rigorous benchmarking against established algorithms in the specific context of the AGC tuning problem.

To bridge these identified gaps, this paper proposes a novel, carefully justified fractional-order cascade controller, designated as the (1 + FOPI)-FOPI-TID, specifically tailored for automatic generation control of two-area power systems. The proposed controller simultaneously utilizes the area control error (ACE) and frequency deviation (*Δ F*) signals, incorporating fractional-order dynamics and a tilting integral block $s^{{ - 1_{{/_{n} }} }}$ to enhance transient response, disturbance rejection, and robustness^29^. Optimal parameter tuning is achieved using the weighted average algorithm (WAA)^30^. The straightforward yet efficient search process of the Weighted Average Algorithm (WAA), which is based on deterministic weighted average of potential solutions based on their fitness scores, sets it apart from other metaheuristic techniques. WAA avoids complicated population dynamics and requires fewer control parameters than evolutionary and swarm-based algorithms, which rely on several stochastic operators and algorithm-specific parameters. WAA is ideally suited for controller tuning in large-scale interconnected power systems because the adaptive weighting technique allows for a natural balance between exploration and exploitation, leading to quick convergence, numerical robustness, and less computational effort.

In accordance with the “no-free-lunch” principle^31^, the dual exploration–exploitation capability of WAA ensures robust regulation in the presence of GRC, GDB, BD, CTD, and stealthy cyber threats. Its novelty lies in the dynamic adjustment of search space via weighted population positions, which enhances convergence stability.

The Contributions of this study are summarized as follows:

i. A novel tri-stage FO CC, designed as (1 + FOPI)-FOPI-TID, is proposed for AGC of PSI to enhance frequency regulation and tie-line power stability.
ii. The proposed controller employs dual input signals, *ACE* and *Δ F*, enabling faster disturbance rejection and improved transient performance compared to conventional single-input CC structures.
iii. The WAA, a recent metaheuristic algorithm, is utilized for the optimal tuning of the proposed controller parameters, providing an effective balance between exploration and exploitation and ensuring robust convergence characteristics.
iv. A realistic multi-source two-area power system model is developed by incorporating GRC, GDB, BD, and CTD nonlinearities.
v. The cyber-resilience of the proposed LFC scheme is explicitly investigated by subjecting the system to resonance-based cyber-attack scenarios, assessing frequency deviation and rate-of-change-of-frequency (RoCoF) performance.

## Materials and methods

### Modelling of hybrid power system

**Fig. 1:**
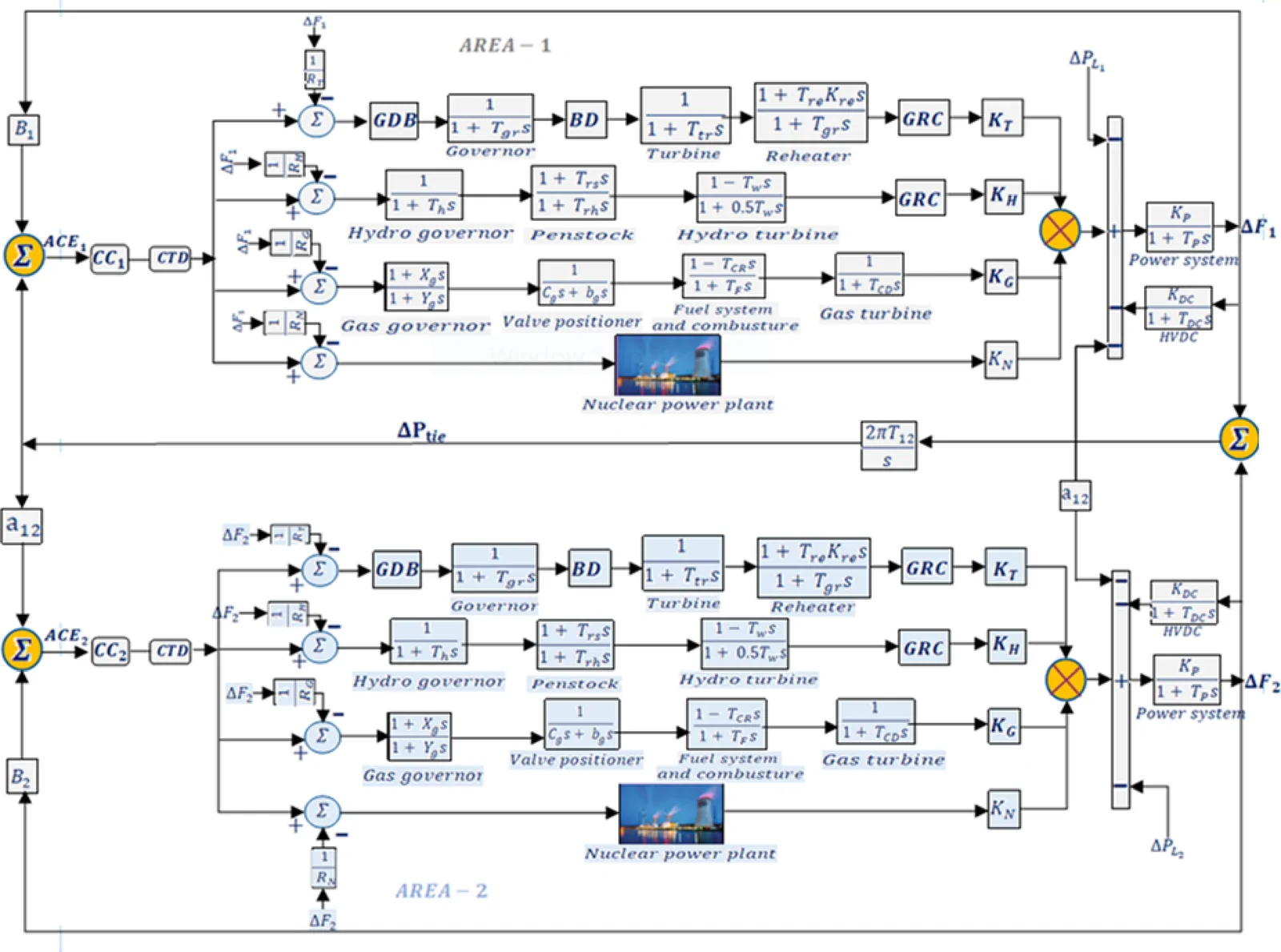
Transfer function model of the tested PSI.

The investigated system in Fig. 1. is a two-area interconnected power system with multi-source generation in each control area. Area-1 consists of thermal reheat, hydro, gas, and nuclear power generation units, while Area-2 comprises an identical combination of generation sources.

The power demand is represented in a step load form, with an increment of $\Delta {P}_{l1}$ for area1 and $\Delta {P}_{l2}$ for area-2. The system includes two frequency variations ${\Delta F}_{i}$, ${\Delta F}_{1}$ for area-1 and ${\Delta F}_{2}$ for area-2, and tie-line power change $\Delta {P}_{tie}$. Frequency regulation is achieved through feedback mechanisms utilizing the speed regulation (*R*) and frequency bias factors (*B*), which stabilize system dynamics by adjusting tie-line power and frequency deviations^32^.

The effect of combining tie-line power error and frequency fluctuation is attributed to the ACE^17,33^. The area-specific ACEs are:

1 $$\begin{aligned} ACE_{1} = & \Delta P_{tie} + B_{1} \Delta F_{1} \\ ACE_{2} = & \Delta P_{tie} + B_{2} \Delta F_{2} \\ \end{aligned}$$

In the event of a load disturbance on the PS, ACEs are employed as a regulating signal to reduce $\Delta {P}_{tie}$ and ${\Delta F}_{\mathrm{i}}$ to zero at the steady state.

The integration of the HVDC link in the PSI allows for additional investigation. The generation rates from thermal, hydro, gas, and nuclear units are managed by independent secondary controllers in each sector, with AC-DC parallel lines connecting these units, as illustrated in Fig. 1. The DC link is modeled using a first-order transfer function, characterized by a gain of ${\mathrm{K}}_{\mathrm{DC}}$ and a time constant of ${\mathrm{T}}_{\mathrm{DC}}$. This first-order transfer function effectively describes the behavior of the DC link.

2 $$\Delta {P}_{\mathrm{tie,DC}}=\frac{{\mathrm{K}}_{\mathrm{DC}}}{1+{\mathrm{T}}_{\mathrm{DC}}\mathrm{s}}$$

The HVDC links are increasingly being utilized for power exchange across control areas alongside existing high-voltage AC links. Research indicates that HVDC links, when operating in tandem with conventional AC systems, provide cost-effective and environmentally friendly benefits while also ensuring higher-quality electric power delivery^34^. Moreover, it reinforces the system’s resistance to minor disruptions^35^.

### Nonlinear test models

**Fig. 2:**
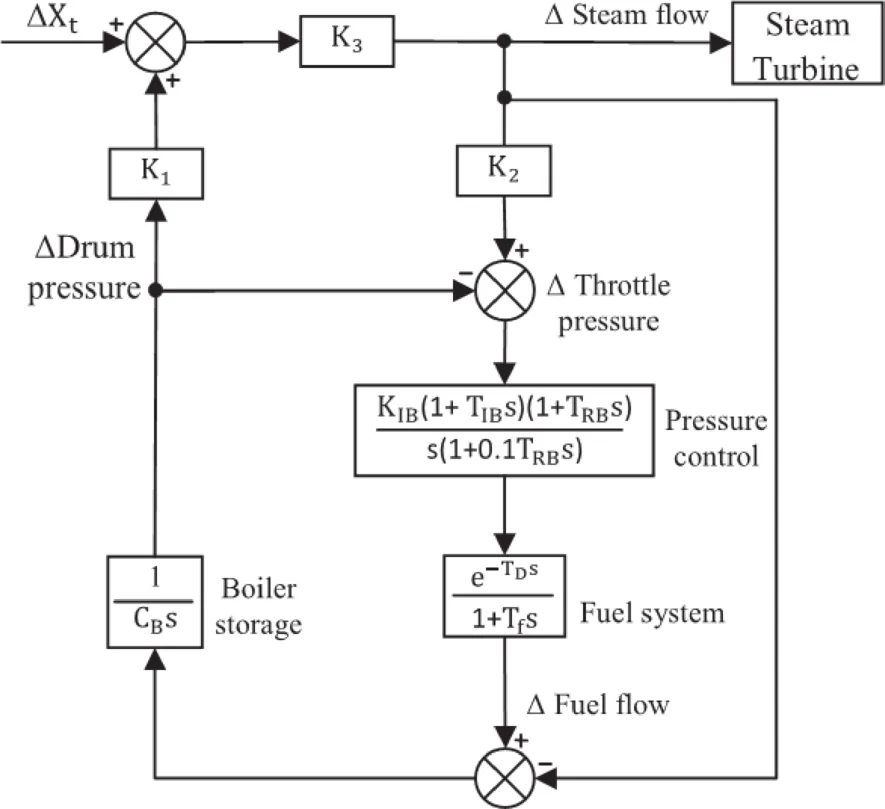
Boiler dynamic nonlinearity.

Several physical nonlinearities are incorporated into the test model of the PSI to enhance realism. As shown in Figs. 1 and 2, the test model includes nonlinearities such as GDB, GRC, BD, and CTD. To limit the rate of generation change, a significant nonlinear component known as GRC is added. The typical benchmark for thermal plants is a GRC value of 3% per minute. To prevent excessive generation, the GRC for the thermal system is specified as $\left|\Delta {P}_{g}\right|$≤*0.0005 p.u.MW/s*. To efficiently control generation levels, the saturation block of the thermal unit is restricted to ± 0.0005. A GRC of 360% per minute for decreasing generation and 270% per minute for increasing generation was taken into consideration for hydropower plants. This means that for decreasing generation, |ΔP_g_(s)|≤ 0.06 p.u.MW/s, and for increasing generation, |ΔP_g_(s)|≤ 0.045 p.u.MW/s. According to^36^ and^37^, the system model includes a saturation block in the hydro turbine that is limited between -0.06 and 0.045. According to Gheisarnejad M.^38,39^, the GDB nonlinearity is spelled out as:

3 $${G}_{\mathrm{d}}\left(s\right)=\frac{0.8-\frac{0.2}{\pi }s}{1+{T}_{\mathrm{g}}s}$$

**Fig. 3:**
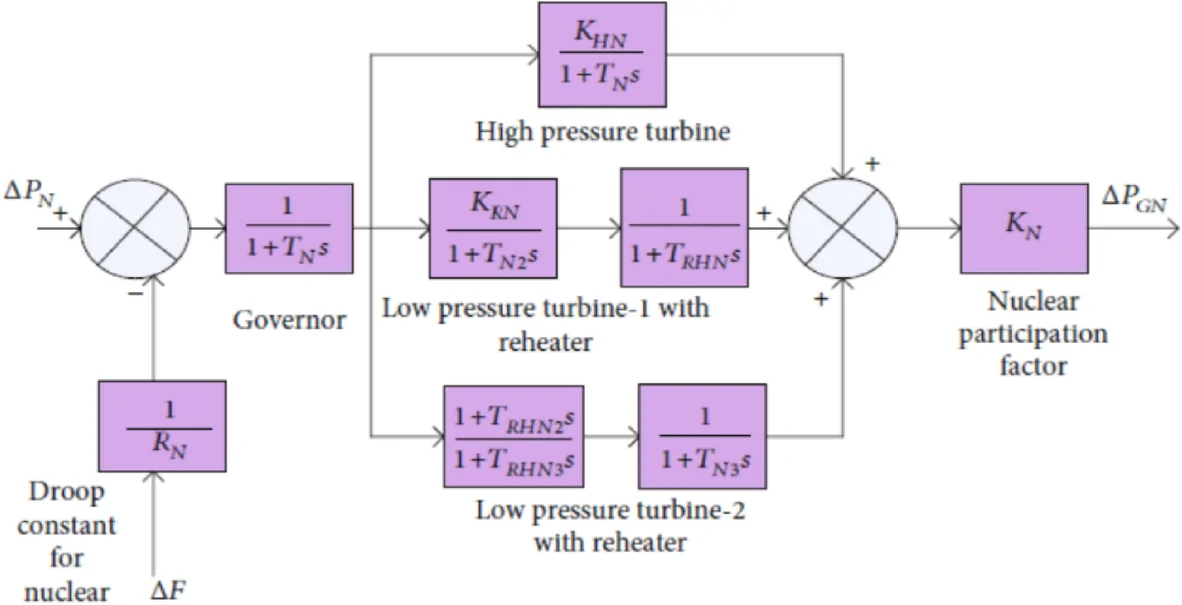
Nuclear power plant transfer function model.

Figure 3. presents a simplified dynamic model of a nuclear power plant for load frequency control analysis. It includes the governor, high- and low-pressure turbine stages with reheater dynamics, and a nuclear participation factor. Frequency deviation is processed through droop and governor dynamics, while turbine-reheater blocks capture the slow response of nuclear units. The aggregated turbine output determines the incremental nuclear power contribution, enabling assessment of nuclear participation in frequency regulation under load disturbances.

Similarly, a boiler dynamic nonlinearity is added to the thermal reheat system, as illustrated in Fig. 2. ^11,40^. The figure presents a dynamic boiler-steam turbine model used in load frequency control studies of thermal power plants, capturing the interactions among the boiler, fuel system, pressure control loop, and steam turbine. The control input signal regulates steam flow to the turbine through appropriate gain factors, while feedback from drum and throttle pressure deviations ensures stable operation. Boiler dynamics are represented using a storage model, and the pressure control loop incorporates proportional-integral with lead-lag characteristics. The fuel system is modeled as a first-order process with time delay to account for fuel transport and combustion effects. Overall, the model effectively represents the dominant delayed and nonlinear dynamics of thermal units for frequency regulation analysis under load disturbances.

Additionally, a CTD is incorporated after the controller design to enhance the realism of the system. According to^41^ and ^42^, this study used a time delay parameter (${\uptau }_{d}$) value of 0.25 s, which is normally in the [0:1] range:

4 $${e}^{-s{\uptau }_{d}} = \frac{1-\frac{{\uptau }_{d }}{2}\mathrm{s}}{1+\frac{{\uptau }_{d }}{2}\mathrm{s}}$$

### Cyber attack

Cyber-attacks in LFM are increasingly important to consider in order to ensure the reliable and secure operation of modern PSs, as such attacks may disrupt control actions and lead to severe operational consequences. In this work, a resonance-based cyber-attack (ResA) is adopted as a physics-informed and literature-supported representation of stealthy load manipulation scenarios reported in recent studies on cyber–physical power systems. In a ResA, an attacker introduces load variations that follow a resonance source while remaining within permissible operating limits, thereby avoiding immediate detection by conventional protection mechanisms^9,43^. Although these variations are small in magnitude, their persistent nature can induce pronounced frequency deviations and elevated RoCoF, which may threaten system stability.

The attack parameters, including amplitude and frequency, are selected based on established literature to reflect realistic adversarial behavior rather than idealized worst-case conditions. This choice ensures that the injected disturbances remain credible and allows a fair comparative assessment, as all controllers are evaluated under identical attack conditions. In practical power systems, RoCoF-based protection relays are widely deployed to mitigate such disturbances. Typical RoCoF relay thresholds lie within the range of 0.1–1.0 Hz/s, depending on system inertia characteristics^9^, and exceeding these limits may trigger protective actions that result in major system disruptions. Under the considered ResA, the manipulated load signal is verified for plausibility by the power generation system; once accepted, the attacker can influence system dynamics in a sustained manner^9,43^.

In this study, RoCoF is employed as the primary cyber-resilience indicator due to its direct relevance to frequency protection schemes and operational security in interconnected power systems. While additional cyber-resilience metrics, such as observability degradation and control signal distortion, are recognized as important complementary indicators, their detailed investigation is beyond the scope of the present work and is identified as a direction for future research. The mathematical representation of the resonance-based cyber-attack is given by:

5 $$\Delta {P}_{L2}= -0.1.sign\left[\Delta {f}_{2}{\prime}\right]$$

which relates the injected load disturbance to the frequency variation in the second control area, consistent with previously reported models^43^.

### Design of proposed (1 + FOPI)-FOPI-TID controller structure

**Fig. 4:**
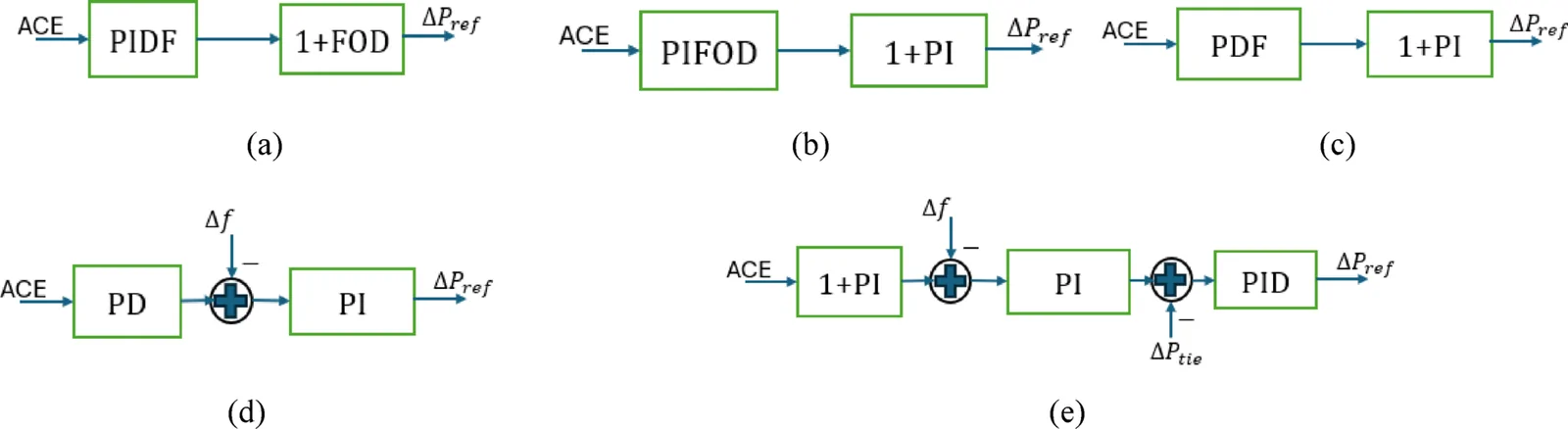
The literature controller structure: (**a**) PIDF-(1 + FOD), (**b**) PIFOD-(1 + PI), (**c**) PDF-(1 + PI), (**d**) PD-PI, and (**e**) (1 + PI)-PI-PID.

As shown in Table 1, the literature controller structures include two-stage configurations such as PIDF-(1 + FOD), PIFOD-(1 + PI), and PDF-(1 + PI), where the *ACE* is the sole input. In contrast, the PD-PI controller utilizes both *ACE* and *Δ F* as inputs; however, *Δ F* is directly fed into the secondary PI controller, which limits disturbance rejection. Additionally, the tri-stage (1 + PI)-PI-PID controller incorporates *ACE*, *Δ F*, and $\Delta {P}_{\mathrm{tie}}$, but retains a conventional PID structure, with $\Delta {P}_{\mathrm{tie}}$ passing through only one controller, the PID. These controller structures are shown in Fig. 4. A comparative analysis of controller attributes provided in terms of controller structure, number of stages, scale of PSI, and limitations and advantages are summarized in Table 1.

**Table 1:** Controller structure, scale, input, limitations, and advantages.

Ref	Scale	CC structure	Input Signals	Limitations	Advantages
^44^	Maritime microgrid	PI-(1 + PD)	*Δ F*	- PID controller and conventional structure - It depends on ACE or $\Delta \mathrm{F}$ only	- Reduces high-frequency noise better than single PID - Better stability and faster disturbance rejection
^45^	Two-area hydrothermal IPS in deregulated environment	PIDF-(1 + FOD)	ACE		
^46^	Three-area system with GRC &FACTS	PDF + (1 + PI)	ACE		
^47^	Two-area of different test models	PD-PI	ACE, and *Δ F*	-$\Delta \mathrm{F}$ only passes to PI controller, losing disturbance rejection since it does not pass through two controllers - Using PID controller	- Fast stabilization with minimal peaks - Enhances robustness and flexibility in high-variability conditions
^25^	Two area: reheat, thermal, and hydropower PSI	(1 + PI)-PI-PID	ACE, *Δ F* and $\Delta {P}_{\mathrm{tie}}$	- Using PID controller -$\Delta {\mathrm{P}}_{\mathrm{tie}}$ only passes to PID controller, losing disturbance rejection as it does not pass through two controllers	
^23^	Two-area microgrid with tidal PS	PIFOD-(1 + PI)	ACE	-Tested on small PSs and one input ACE	- Cascade controller attain good transient response

**Fig. 5:**
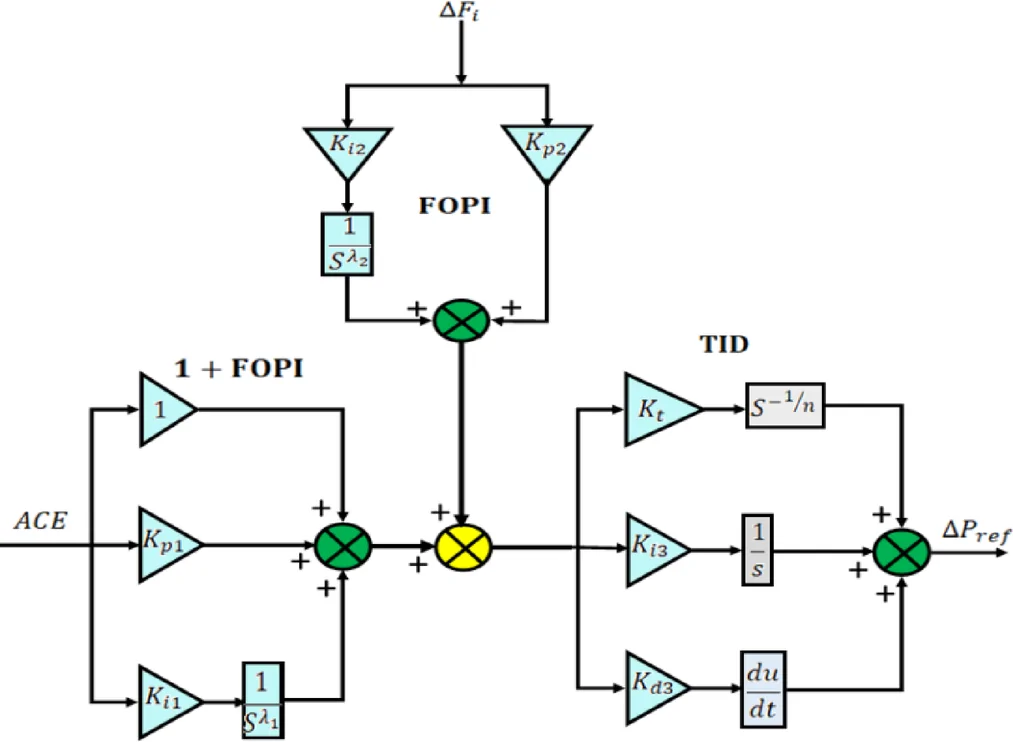
Proposed structure of (1 + FOPI)-FOPI-TID CC.

This paper introduces a novel controller structure of (1 + FOPI)-FOPI-TID, as illustrated in Fig. 5. This configuration allows for the swift rejection of disturbance sources before they can propagate through the system. The unique controller incorporates two input signals: the main *ACE* and *Δ F*. This feature distinguishes it from other CC configurations in the field, as the *ACE* serves as the primary input to the (1 + FOPI) controller, in parallel, the *Δ F* is the input to the FOPI controller, and the combined output of both feeds into the TID controller.

Although the *ACE* already contains frequency deviation information ($ACE={\Delta P}_{tie}+ {\Delta F}_{i}$ ), using both ${\Delta F}_{i}$ and *ACE* as inputs in the proposed controller is intentional and meaningful. The two signals represent different aspects of system behavior. The frequency deviation ${\Delta F}_{i}$ reflects fast local changes caused by sudden load disturbances and is useful for taking quick corrective action. On the other hand, the *ACE* signal represents the overall control objective, as it includes both frequency deviation and tie-line power deviation, and is responsible for coordinating power exchange between areas and restoring steady-state operation. By assigning ${\Delta F}_{i}$ and *ACE* two different stages of the CC, the proposed structure separates fast local response from slower inter-area regulation. Even though these signals are mathematically related, they act on different time scales, and their combined use does not lead to conflicting control actions. Instead, this structured design improves disturbance rejection, damping, and robustness, especially in the presence of nonlinearities and uncertainties.

Therefore, the proposed controller is not unnecessarily complex but rather designed to handle the practical dynamic characteristics of interconnected power systems more effectively. The S-domain representations of the (1 + FOPI), FOPI, and TID controllers are as follows:

6 $${G}_{1+FOPI}\left(\mathrm{s}\right)=1+{K}_{p1}+ {K}_{i1}{s}^{-\lambda 1}$$

7 $${G}_{FOPI}\left(\mathrm{s}\right)={K}_{p2}+ {K}_{i2}{s}^{-\lambda 2}$$

8 $$G_{{TID}} \left( s \right) = K_{t} s^{{ - 1_{{/_{n} }} }} + \frac{{K_{{i3}} }}{S} + K_{d} s$$

where ${K}_{pi}$, ${K}_{i}$,${K}_{t}$, ${K}_{d}$ are proportional, integral, tilt, derivative, while *λ*, and n are the fractional parameters of integral and tilt factors. Achieving optimal performance for this controller requires the simultaneous optimization of these ten parameters. To fulfill this objective and attain fair comparison, a WAA is selected for this study to tune the proposed and the literature controller structures.

In this study, The FO operators were realized using the Oustaloup recursive approximation method, which provides accurate finite‑dimensional representations of fractional differ‑integrators over the selected frequency band [0.01–100] rad/s. This choice ensures numerical stability and efficient implementation in MATLAB/Simulink. The controller parameters are optimized offline, and the resulting implementation requires only low-order filters and simple algebraic operations, making the proposed control scheme computationally efficient and suitable for real-time deployment in large-scale interconnected power systems, even in the presence of communication delays.

The proposed (1 + FOPI)–FOPI–TID CC architecture is motivated by the physical structure and different response time requirements of automatic generation control rather than mathematical complexity alone. Each stage is assigned a distinct control role: the (1 + FOPI) loop, driven by the area control error, ensures accurate steady-state regulation and coordinated inter-area power exchange, with fractional-order integration enhancing robustness to uncertainties and low-frequency oscillations. The FOPI loop directly processes local frequency deviation, enabling rapid disturbance rejection and improved damping under sudden load changes and nonlinear operating conditions. The final TID stage functions as a dynamic compensator, where the tilt component provides wide-band damping, the integral term removes residual steady-state offsets, and the derivative term improves transient shaping without excessive noise amplification. This cascade configuration deliberately separates fast local frequency control from slower inter-area regulation, reducing control interaction and avoiding aggressive gain tuning required in simpler single- or dual-loop fractional controllers. As a result, the proposed structure closely reflects the physical control objectives of interconnected power systems, thereby enhancing robustness, interpretability, and practical applicability beyond purely performance-based validation.

### Performance index of LFM

Customer demand changes affect the PS’s frequency, which causes $\Delta {F}_{i}$ and $\Delta {P}_{tie}$ to deviate from their nominal values. With an appropriate Performance function, it is advised to first successfully use contemporary heuristic optimization-based controller techniques in order to improve LFM performance^39,48,49^. The ISE, ITAE, IAE, and ITSE are some of the common performance functions that have been literature to address AGC issues and restore nominal performance^43,50^.

The ITAE criterion was chosen as the primary objective because of its proven effectiveness in minimizing sustained deviations and improving damping in AGC studies, thereby ensuring faster convergence and reduced steady‑state error^51^. ITAE provides a balanced trade‑off between transient suppression and long‑term stability, which aligns with the focus of this work. The application of the ITAE criterion will be essential in this investigation in order to modify the (1 + FOPI)-FOPI-TID CC’s scaling parameters. This objective is represented by the symbol ξ as follows:

9 $$\xi =\mathrm{ITAE}={\int }_{0}^{{\mathrm{t}}_{\mathrm{sim}}}\mathrm{t}\cdot \left[{\Delta F}_{1} +{\Delta F}_{2} +\Delta {P}_{\mathrm{tie}}\right]\mathrm{dt}$$

In order to find out optimistic controller gains on a large scale, the gains range are chosen in the range $\left[0, 2\right]$ as in^24,36^ for non-linear MSTA model. The n and λ in the range $\left[2, 3\right]$ ^52,53^ and $\left[0, 2\right]$ ^11^ respectively. In conclusion, by exploiting the WAA metaheuristic algorithm, optimal or near optimal set of (1 + FOPI)-FOPI-TID CC parameters is derived within the recommended edges for the smallest value of *ξ .*

## Weighted average algorithm (WAA)

The two main steps of population-based meta-heuristic algorithms are exploitation and exploration. Exploration is concerned with avoiding local optima in the search space (SS), whereas exploitation is concerned with bettering solutions found during exploration. This approach is best shown by the WAA, which strikes a balance between exploration and exploitation. To keep this equilibrium, it uses movement strategies based on random constants and iteration numbers and calculates the population’s weighted average position (WAP) at each iteration.

### The weighted average position’s inspiration

Each element is given a certain weight in order to calculate the WAP; the final average is more affected by the higher weights. The SS directs the updating of each person’s location for the subsequent iteration during each iteration. Individual positions, their personal best position (PBP), and the global best position (GBP) form the basis of the new SS. The global position is the best fitness value for the entire population, whereas a person’s PBP is the maximum fitness value attained during its movement history. Together with the PBP and GBP, the WAP is used to update future positions and represents the current population distribution.

### Algorithm and mathematical model

Matrix S, which represents a collection of potential solutions, is used to randomly begin the optimization process:

$$S=\left[\begin{array}{c}\begin{array}{c}{S}_{1}\\ {S}_{2}\end{array}\\ \begin{array}{c}\vdots \\ {S}_{i}\\ \vdots \end{array}\\ {S}_{n}\end{array}\right]=\left[\begin{array}{cccccc}{s}_{1}^{1}& {s}_{1}^{2}& \cdots & {s}_{1}^{j}& \cdots & {s}_{1}^{d}\\ {s}_{2}^{1}& {s}_{2}^{2}& \cdots & {s}_{2}^{j}& \cdots & {s}_{2}^{d}\\ \vdots & \vdots & \cdots & \vdots & \cdots & \vdots \\ {s}_{i}^{1}& {s}_{i}^{2}& \cdots & {s}_{i}^{j}& \cdots & {s}_{i}^{d}\\ \vdots & \vdots & \cdots & \vdots & \cdots & \vdots \\ {s}_{n}^{1}& {s}_{n}^{2}& \cdots & {s}_{n}^{j}& \cdots & {s}_{n}^{d}\end{array}\right], \mathrm{where} \left\{\begin{array}{c}i=1, 2, \dots ,n.\\ j=1, 2, \dots ,d.\end{array}\right.$$

where *d* is the dimension of the deemed problem, $n \text{is the number}$ of solutions candidate along the SS, and ${S}_{i}$ is the ${i}^{th}$ candidate solution, , ^30^. The starting positions ${s}_{i}^{j}$ are disclosed randomly as:

$${s}_{i}^{j}={LB}_{j}+rand.\left({UB}_{j}-{LB}_{j}\right)$$

where ${LB}_{j}$ refer to the ${j}^{th}$ lower bound value, *rand* is a random number within [0: 1] range, and ${UB}_{j}$ expresses the ${j}^{th}$ upper bound value.

#### Weighted average position (WAP)

First, each participant’s fitness is determined, then the population is rearranged according to the cost function to determine the WAP. The first N candidates in the population are then chosen in order to compute the WAP in the manner described below:

$${N}_{candidate}=\left(nP-4\right)\frac{it-1}{1-{Max}_{It}}+nP$$

$${SUM}_{Fitness}=\sum_{i=1}^{{N}_{candidate}}Fitness ({S}_{i})$$

$${X}_{miu}=\frac{\sum_{i=1}^{{N}_{candidate}}{S}_{i}({SUM}_{Fitness}-Fitness \left({S}_{i}\right))}{{SUM}_{Fitness}({N}_{candidate}-1)}$$

where ${S}_{i}$ is the *i*^*th*^ candidate solution, ${SUM}_{Fitness}$ is the total fitness values of the chosen candidates, *nP* is the populations number, *Fitness* is the function used to calculate the fitness value, ${N}_{candidate}$ is the number of chosen candidate solutions, ${N}_{candidate}$ is the applicant solutions number, ${X}_{miu}$ is WAP. Besides, *it* is the current iteration number, and ${Max}_{It}$ is the limit number of iterations.

- Exploitation or exploration process

The search phase for the potential solution, i.e., exploration or exploitation, should be identified during the iteration process:

$$f\left(it\right)=\left(\alpha .rand-1\right)\mathrm{sin}\left(\pi \frac{it}{{Max}_{It}}\right)$$

The balance between exploration and exploitation phases is controlled by the constant *α*. An equilibrium is reached if $f\left(it\right)= 0.5$. Candidate solutions with $f\left(it\right)\ge 0.5$ move in accordance to their exploitation capabilities, whereas those with $f\left(it\right)<0.5$ move in accordance to their exploration abilities^30^

(b) Exploitation phase

**Fig. 6:**
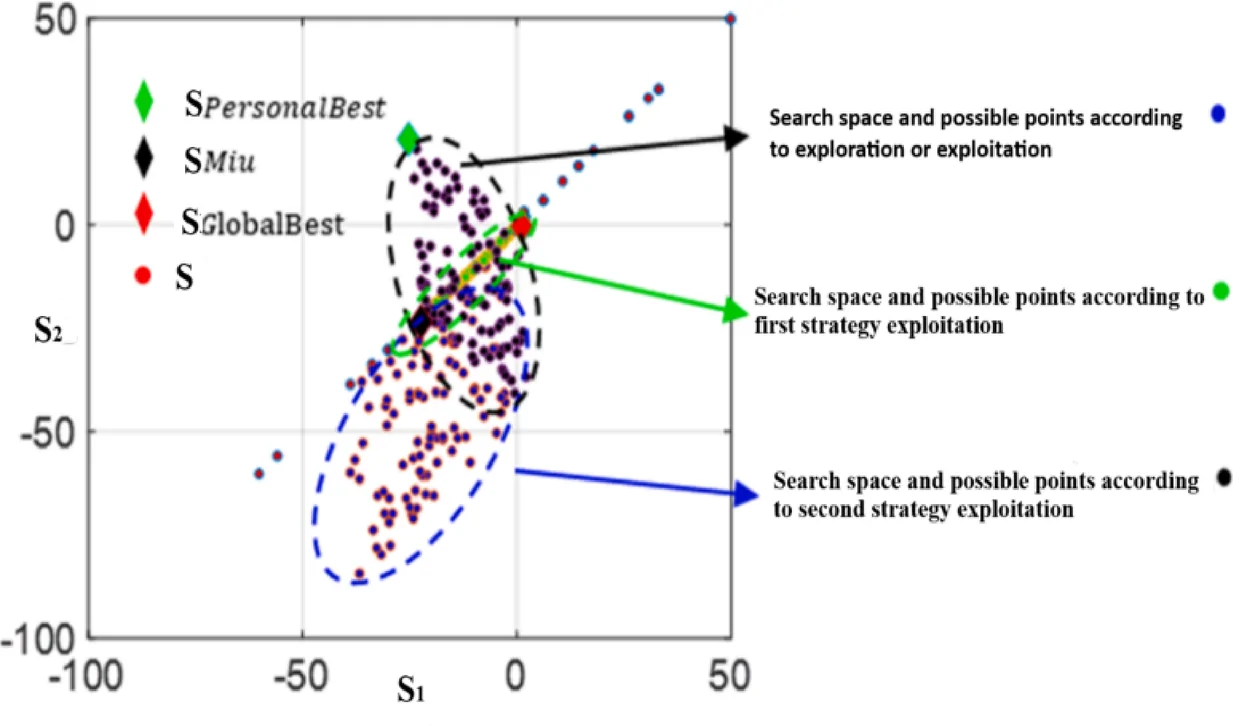
Diagram of search agent position updating based on exploitation strategies.

The exploitation strategy in the WAA directs search agents towards areas likely to yield new global best values. To efficiently exploit the SS, the WAA employs three movement strategies that incrementally move search agents closer to the optimal solution as shown in Fig. 6.

(i) The first strategy exploits the SS using the WAP of the entire population, PBP, and GBPs.

$${S}_{i}\left(it+1\right)={r}_{11}.\left({S}_{miu}\left(it\right)-{S}_{G{lobal}_{best}}\left(it\right)\right)+{r}_{12}.\left({S}_{miu}\left(it\right)-{S}_{{Personal}_{best}}\left(it\right)\right)+{r}_{13}.{S}_{miu}\left(it\right)$$

where ${r}_{ij}$ are random values within [0–1], to modify the expansion of the search space around the personal best ${S}_{{Personal}_{best}}$ and GBP s ${S}_{G{lobal}_{best}}$ to express the PBP and GBP in the iteration numbers (*it)*.

(ii) The second technique ignores the GBP and concentrates on the region between the PBP and the population’s WAP in order to narrow the SS. This method, in contrast to the first technique, prioritizes a narrower area for exploitation. Using the following, the new SS is produced^34^:

$${S}_{i}\left(it+1\right)={r}_{21}.\left({S}_{miu}\left(it\right)-{S}_{{Personal}_{best}}\left(it\right)\right)+{r}_{22}.{S}_{{Personal}_{best}}\left(it\right)$$

This approach shows a greater bias towards improving accuracy and convergence rate by guiding movement from the agent’s PBP towards the population’s WAP.

(iii) The third trick deals with the SS between the GBP and the population’s WAP. Each candidate’s position update is determined for this purpose using the formula below:

$${S}_{i}\left(it+1\right)={r}_{31}.\left({S}_{miu}\left(it\right)-{S}_{{Global}_{best}}\left(it\right)\right)+{r}_{32}.{S}_{{Global}_{best}}\left(it\right)$$

This approach has a narrow SS that concentrates on the area between the GBP and the WAP. Both the accuracy and the pace of convergence are improved by this focused movement^34^.

(c) Exploration phase

The exploration of the solution space utilizes two strategies: the Levy Flight method and dynamic movement distances. The Levy flight method focuses on the GBP, creating a broad search area around the WAP. This involves short movements for local optimization and longer movements to explore distant regions. Candidates dynamically move in various directions from the GBP, enhancing exploration and balancing the exploitation of current solutions. The Levy flight model follows a Levy distribution, characterized by mostly small strides with occasional larger ones, reflecting natural patterns observed in birds and insects, expressed as^30^:

$$X=\frac{U}{|V{|}^{1/\beta }}, U=normal\left(0, {\sigma }_{u}^{2}\right)\& V=normal\left(0, {\sigma }_{v}^{2}\right)$$

where *X* is the step length of the Levy flight influenced by the parameter β, and Γ stands for the Gamma distribution function. Besides, V and U satisfy normal distributions with standard deviations equal to ${\sigma }_{v}$ and ${\sigma }_{u}$ respectively, and zero mean values. The first exploration technique, which is based on a controlled adjustment of step length X, is as follows:

$${S}_{i,j}\left(it+1\right)={S}_{{Global}_{best}}\left(it\right)+X$$

where ${S}_{i,j}\left(it+1\right)$ denotes the *j* ^th^ position of the *i* ^th^ solution at the next iteration. In certain instances, the algorithm’s global optimum might be located close to the GBP yet too far from the ideal GBP value. When using the first method in such circumstances, the WAA runs the danger of convergent to a local optimum. The second movement method is used to modify the SS in order to reduce this danger^34^:

$${S}_{i}(it+1)={LB}_{min}+rand.\left({UB}_{min}-{LB}_{min}\right)$$

where the lower and upper boundaries’ minimum values are denoted by ${LB}_{min}$ and ${UB}_{min}$, respectively. By using this tactic, the search agents can shift to different locations within the SS in an effort to locate possibly superior areas.

**Fig. 7:**
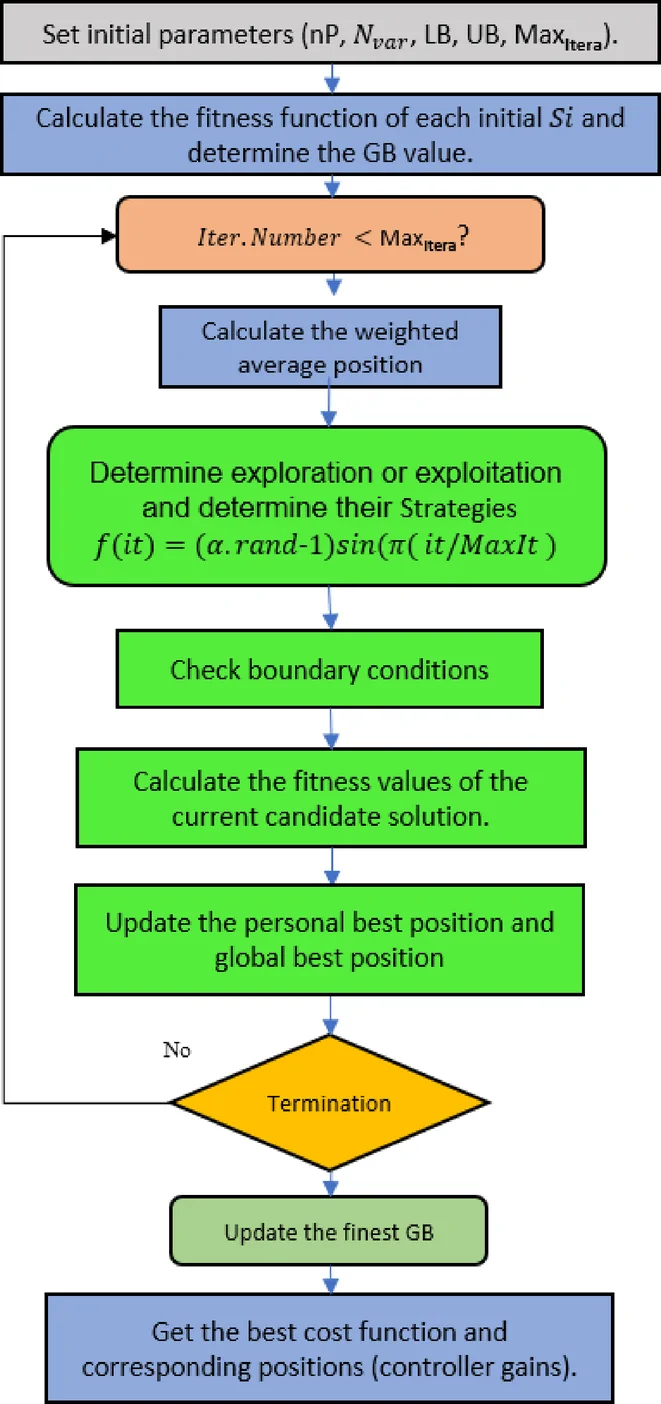
WAA flowchart.

The WAA system optimizes gains in (1 + FOPI)-FOPI-TID controllers using the ITAE criteria. It iteratively generates new gains by minimizing ITAE values, aiming for the lowest ITAE to reduce system deviations. Research indicates that larger initial populations do not necessarily outperform smaller ones in finding optimal solutions, making the approach suitable for real-time LFM scenarios^54^. The operational framework of the proposed WAA is depicted in the flowchart presented in Fig. 7, which outlines the sequential logic and decision-making steps involved in the algorithm.

## Results and discussion

This study investigates the effectiveness of a novel (1 + FOPI)-FOPI-TID controller using a combination of hybrid power sources and non-linearities, GRC, GDB, BD, and CTD, alongside cyber-attacks. To achieve the LFM goal, the controller coefficients are optimized with the WAA optimizer in MATLAB, integrated with Simulink on an Intel Core i-5 processor. The WAA-based controller settings for the cases study after 100 iterations of optimization are conducted. The robustness of the proposed controller is compared to other regulators, including PIDF(1 + FOD), PIFOD-(1 + PI), (1 + PI)-PI-PID CC, PDF-(1 + PI), and PD-PI CCs, all under fixed load changes of 1% SLP. Results from the analyzed multiarea PSI are thoroughly examined in subsequent case studies.

**Case 1: Effectiveness of the WAA algorithm**

**Fig. 8:**
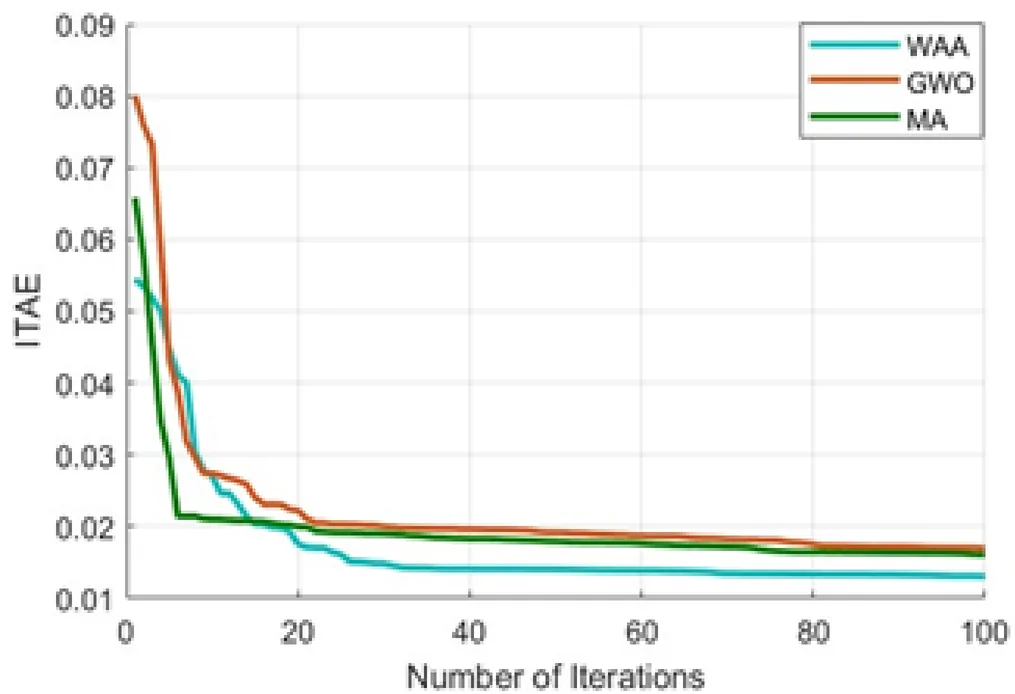
The convergence characteristic curves of PSO, GWO, and the proposed FHO.

To demonstrate the adaptability of the WAA, the convergence characteristics of WAA, mayfly algorithm (MA)^43^, and grey wolf optimization (GWO)^55^ are analyzed in the context of the (1 + FOPI)-FOPI-TID CC using a test model as in Fig. 1. Each optimizer is configured with 100 iterations. The convergence graph of WAA, extracted from MSTA with GDB, GRC, BD, and CTD nonlinearities is depicted in Fig. 8, showcasing WAA’s swift convergence and effectiveness in enhancing AGC control strategies to optimize system performance. Furthermore, a comparison of the minimum, maximum, average, and standard deviation of the ITAE values, ${\xi }_{ITAE}$, in Table 2 illustrates that WAA outperforms MA and GWO in addressing AGC challenges.

**Table 2:** ITAE values for WAA, MA, and GWO at case1.

Algorithm	Minimum	Maximum	Average	St. deviation
WAA	0.013810	0.015443	0.014287	0.00085502
MA	0.016109	0.022811	0.018863	0.00336873
GWO	0.016967	0.023063	0.022399	0.00334436

**Case 2: Analyses of controller’s performance**

**Fig. 9:**
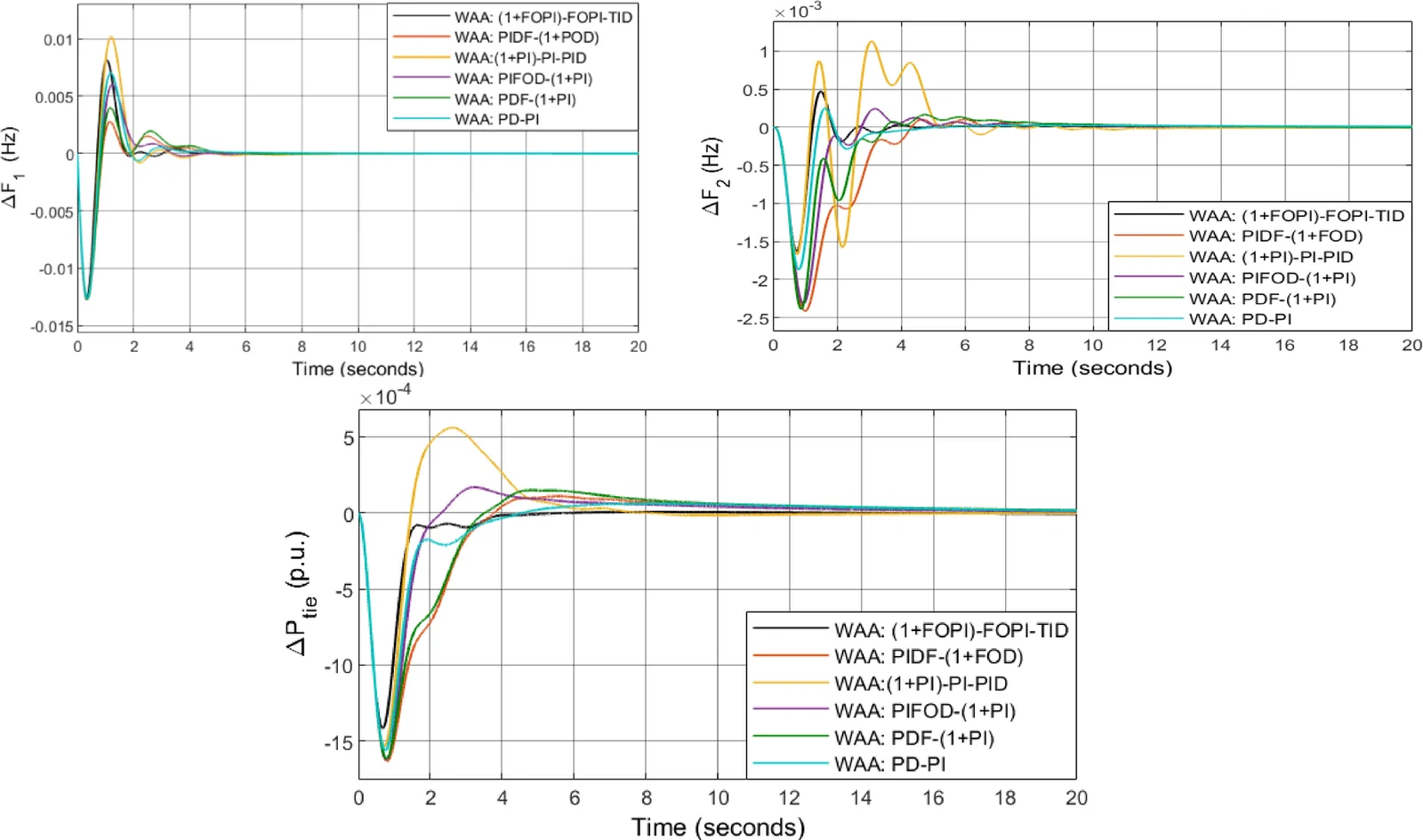
Transient response with various controllers but same optimizer at case 2.

This study conducted a comprehensive performance comparison between the proposed (1 + FOPI)-FOPI-TID controller and various two-stage and three-stage control approaches, including PIDF(1 + FOD), PIFOD-(1 + PI), (1 + PI)-PI-PID CC, PDF-(1 + PI), and PD-PI CC under WAA scheme. The gains of these controllers are listed in Table 3. The evaluation metrics focused on the ITAE value, minimum undershoot (Ush), and settling time (T_s_), considering changes in $\Delta {F}_{1}$*, *$\Delta {P}_{tie}$*,* and $\Delta {F}_{2}$. Overall, Table 4 compares the performance of each optimization procedure using transient metrics. Figure 9 illustrates the dynamic responses of a two-area interconnected power system subjected to a load disturbance, presenting the frequency deviations in Area-1 ($\Delta {F}_{1}$), Area-2 ($\Delta {F}_{2}$), and the tie-line power deviation ( $\Delta {P}_{tie}$) for various WAA-based cascade controller structures. A comparative evaluation is carried out between the proposed WAA-(1 + FOPI)–FOPI–TID controller and other existing controllers, including PIDF-(1 + FOD), (1 + PI)–PI–PID, PIFOD-(1 + PI), PDF-(1 + PI), and PD–PI.

**Table 3:** WAA optimized controller gains under 1% SLP in area 1.

WAA based CC structure	Area 1							Area 2						
	${{\boldsymbol{K}}}_{{\boldsymbol{P}}}/{{\boldsymbol{K}}}_{{\boldsymbol{t}}}$	${{\boldsymbol{K}}}_{{\boldsymbol{I}}}$	${{\boldsymbol{K}}}_{{\boldsymbol{D}}}$	${\boldsymbol{\mu}}$	${\boldsymbol{\uplambda}}$	${\boldsymbol{n}}$	${\boldsymbol{N}}$	${{\boldsymbol{K}}}_{{\boldsymbol{P}}}/{{\boldsymbol{K}}}_{{\boldsymbol{t}}}$	${{\boldsymbol{K}}}_{{\boldsymbol{I}}}$	${{\boldsymbol{K}}}_{{\boldsymbol{D}}}$	${\boldsymbol{\mu}}$	${\boldsymbol{\uplambda}}$	${\boldsymbol{n}}$	${\boldsymbol{N}}$
*PIDF (1 + FOD)*														
PIDF	0.6333	2.0000	1.3e-4	–	–	–	–	1.5019	0.1495	2.0000	–	–	–	–
(1 + FOD)	–	–	–	0.2611	–	–	23.53	–	–	–	1.0e-6	–	–	0.0126
*WAA: PIFOD-(1 + PI)*														
PIFOD	0.6583	2.0000	7.02e-05	0.9757	–	–	–	2.0000	0.2769	0.3816	0.3802	–	–	–
(1 + PI)	2.0000	1.129e-7	–	–	–	–	–	0.0527	0.00373	–	–	–	–	–
*PD-PI CC*														
PD	2.0000	–	0.4935	–	–	–	–	2.0000	–	2.0000	–	–	–	–
PI	0.2709	1.9308	–	–	–	–	–	0.0569	0.0873	–	–	–	–	–
*(1 + FOPI)-FOPI-TID CC*														
(1 + FOPI)	1.2777	2.0000	–	–	1e-5	–	–	2.0000	0.0786	–	–	1.9977	–	–
FOPI	0.6731	0.0769	–	–	0.9628	–	–	1.0e-4	0.0031	–	–	0.4319	–	–
TID	1.9995	1.6399	0.2435	–	–	2.5071	–	1.0e-6	0.0000	0.0000	–	–	2.999	–
*(1 + PI)-PI-PID CC*														
1 + PI	1.6919	1.8777	–	–	–	–	–	1.6752	1.2e-05	–	–	–	–	–
PI	0.3219	1.9545	–	–	–	–	–	0.3582	1.3421	–	–	–	–	–
PID	0.8823	0.0000	0.1154	–	–	–	–	1.2419	1.0861	0.1967	–	–	–	–
*PDF-(1 + PI)*														
PDF	2.0000	–	0.2135	–	–	–	0.2135	0.0311	–	0.5343	–	–	–	100
(1 + PI)	0.0000	2.0000	–	–	–	–	–	0.0000	2.0000	–	–	–	–	–

**Table 4:** Comparison performance of several controllers for case 2.

WAA based:	*ξ*	ST (0.0002 band) (s)			Undershoot(-ve)		
	ITAE	$\Delta {F}_{1}$	$\Delta {F}_{2}$	*Δ P*	$\Delta {F}_{1}$ $\times {10}^{-3}$	$\Delta {F}_{2}$ $\times {10}^{-3}$	*Δ P* $\times {10}^{-4}$
(1 + FOPI)-FOPI-TID	0.01381	1.707	1.735	1.355	13.0	1.621	14.15
PIFOD-(1 + PI)	0.02526	3.264	3.393	1.723	13.0	2.316	16.22
PD-PI	0.02644	3.494	2.582	2.617	13.0	1.871	15.62
PIDF(1 + FOD)	0.03170	4.460	3.890	2.865	13.0	2.416	16.33
(1 + PI)-PI-PID	0.03390	4.157	4.915	4.177	13.0	1.675	15.34
PDF-(1 + PI)	0.03493	4.661	2.610	2.858	13.0	2.388	16.17
% Reduction	**45.3%**	**47.7%**	**32.8%**	**21.4%**	**—**	**3.2%**	**7.8%**

Significant values are in [bold].

As demonstrated in Fig. 9 and summarized in Table 4, the proposed (1 + FOPI)-FOPI-TID control scheme outperformed all comparative techniques. Table 4 presents a comparative performance evaluation of several WAA-based cascade controllers for Case 2 using key dynamic performance indices, including ITAE, settling time (within a ± 0.0002 band), and maximum undershoot of frequency and tie-line power deviations.

Notably, it achieved an ITAE value of 0.01381—representing a 45.3% improvement over the PIFOD-(1 + PI) controller. The scheme also showed significant enhancements in settling times: 47.7% improvement for $\Delta {F}_{1}$, 21.4% for $\Delta {P}_{tie}$, both compared to PIFOD-(1 + PI) CC, and 32.8% improvement for *Δ* *F₂* (compared to PD-PI CC). While no improvement was observed in the percentage undershoot for $\Delta {F}_{1}$, the proposed controller achieved reductions of 3.2% for $\Delta {F}_{2}$ and 7.8% for $\Delta {P}_{tie}$ when compared to the (1 + PI)-PI-PID CC.

**Fig. 10:**
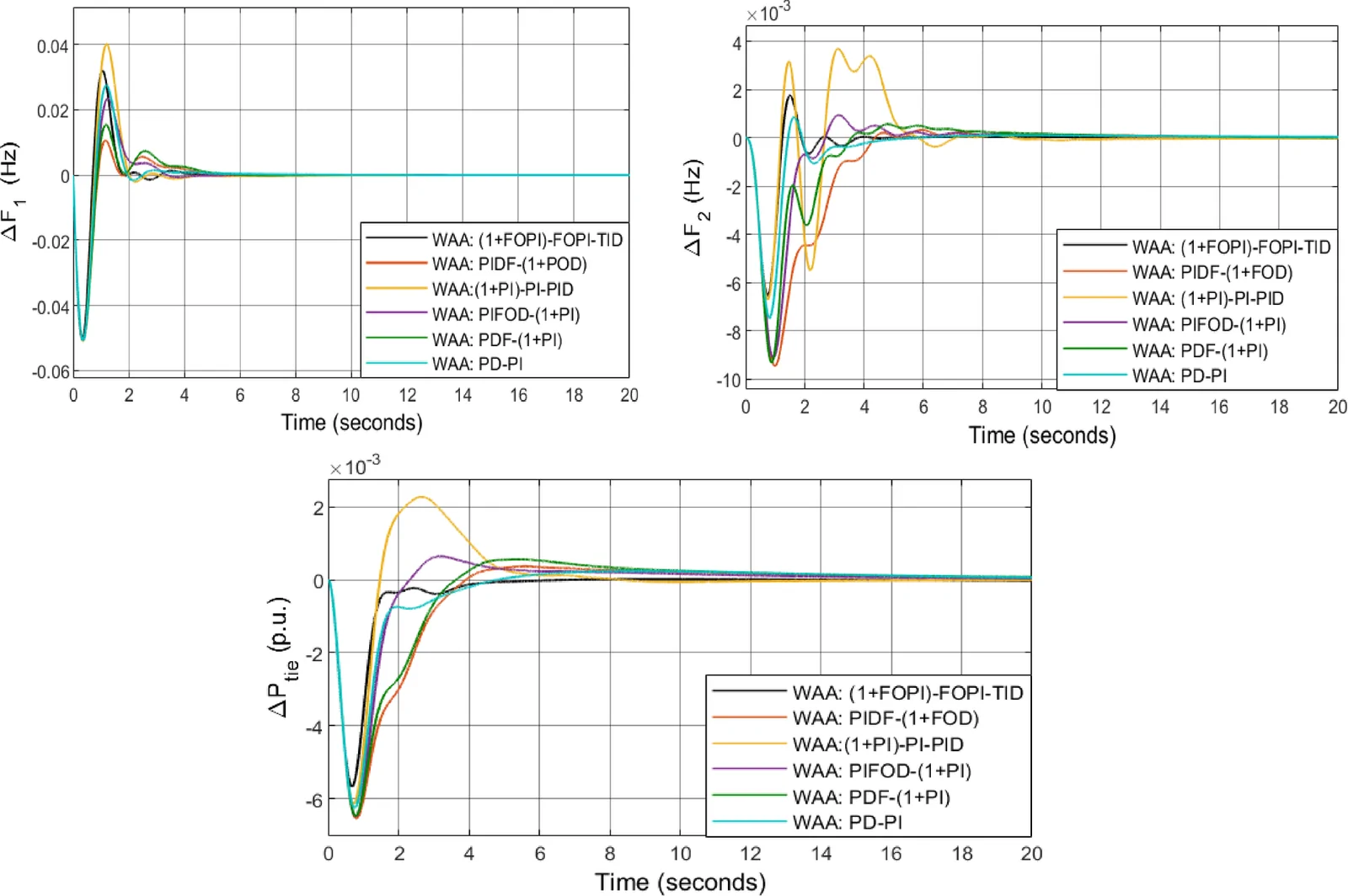
Transient reactions for case 2 with 4% SLC at area 1: (**a**) Δ ${F}_{1}$, (**b**) Δ ${F}_{2}$, and (**c**) ΔP_tie_.

As a result, the proposed (1 + FOPI)-FOPI-TID CC appears to be a potential solution for improving LFM performance in various PSIs. The transient responses of the proposed WAA: (1 + FOPI)-FOPI-TID CC and other controllers were further examined under 4% SLC in area 1, as shown in Fig. 10. The WAA: (1 + FOPI)-FOPI-TID CC outperformed all competing controllers under 4% SLC in area 1, confirming its superiority and demonstrating MSTA stability with nonlinearities.

**Case 3: Performance analyses under random load perturbation**

**Fig. 11:**
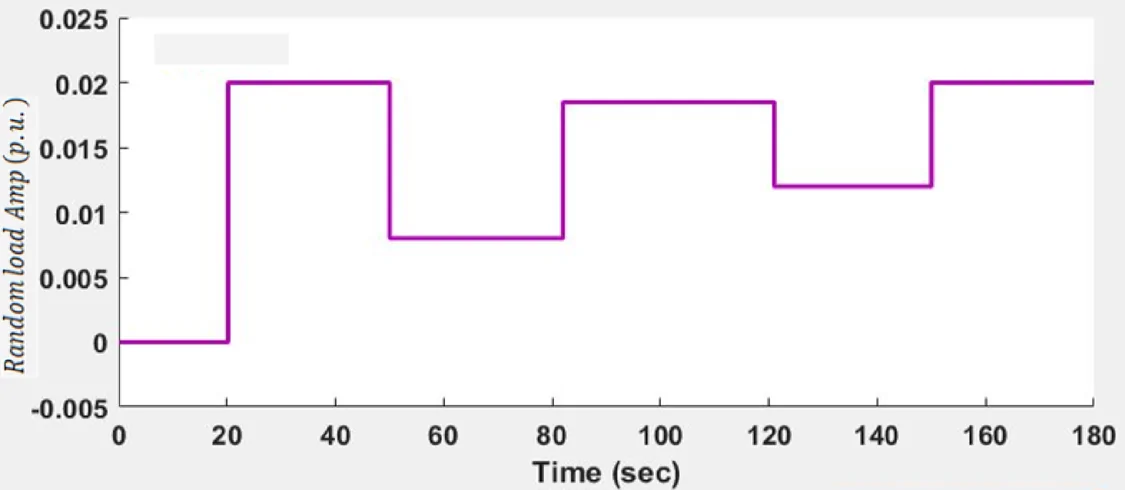
Random step loads ${\Delta P}_{L1}$ in area 1.

**Fig. 12:**
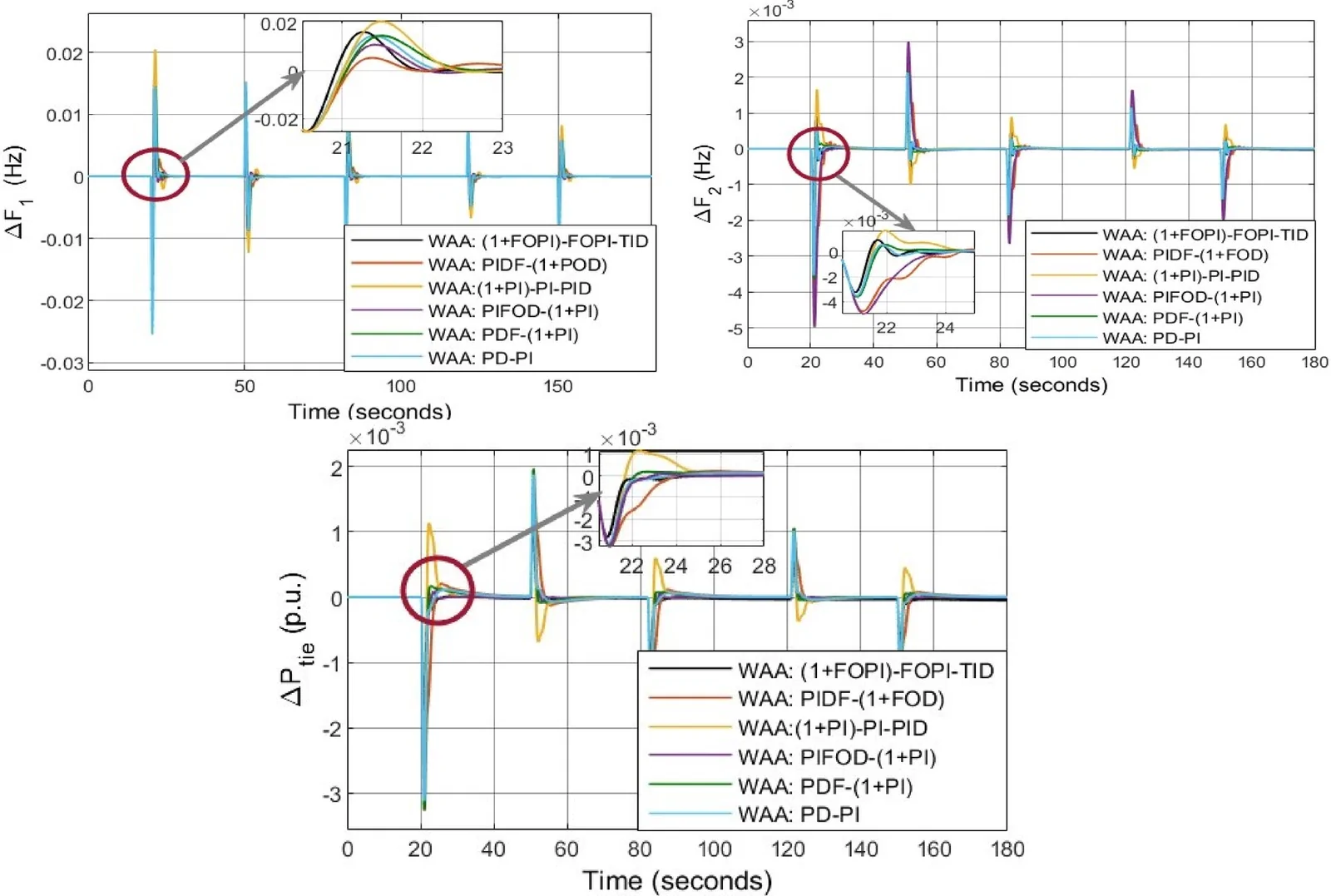
Transient response with various algorithm techniques.

The dynamic analysis of the proposed WAA: (1 + FOPI)-FOPI-TID CC was conducted using a random step load (RSL) in area 1. The RSL perturbation involves a sequence of sudden generating unit outages or abrupt load switches, representing a mix of random load variations as depicted in Fig. 11. Figure 12. depicts the transient responses of a two-area interconnected power system under successive load disturbances, illustrating the frequency deviations in Area-1 ($\Delta {F}_{1}$), Area-2 ($\Delta {F}_{2}$), and the tie-line power deviation ($\Delta {P}_{tie}$) for different WAA-based cascade controller schemes. The highlighted zoomed-in regions emphasize the initial disturbance intervals, allowing a clear comparison of dynamic performance.

**Fig. 13:**
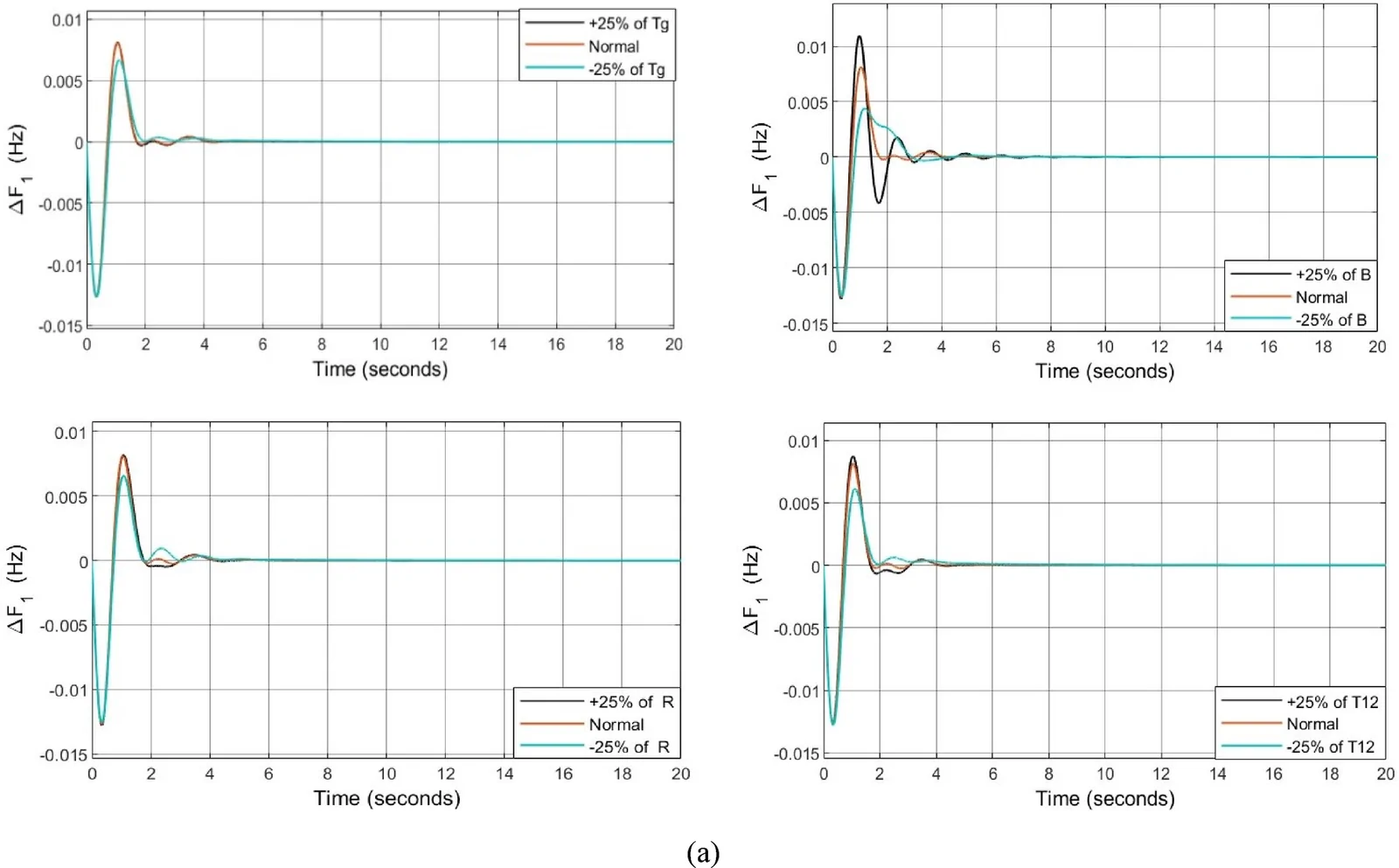

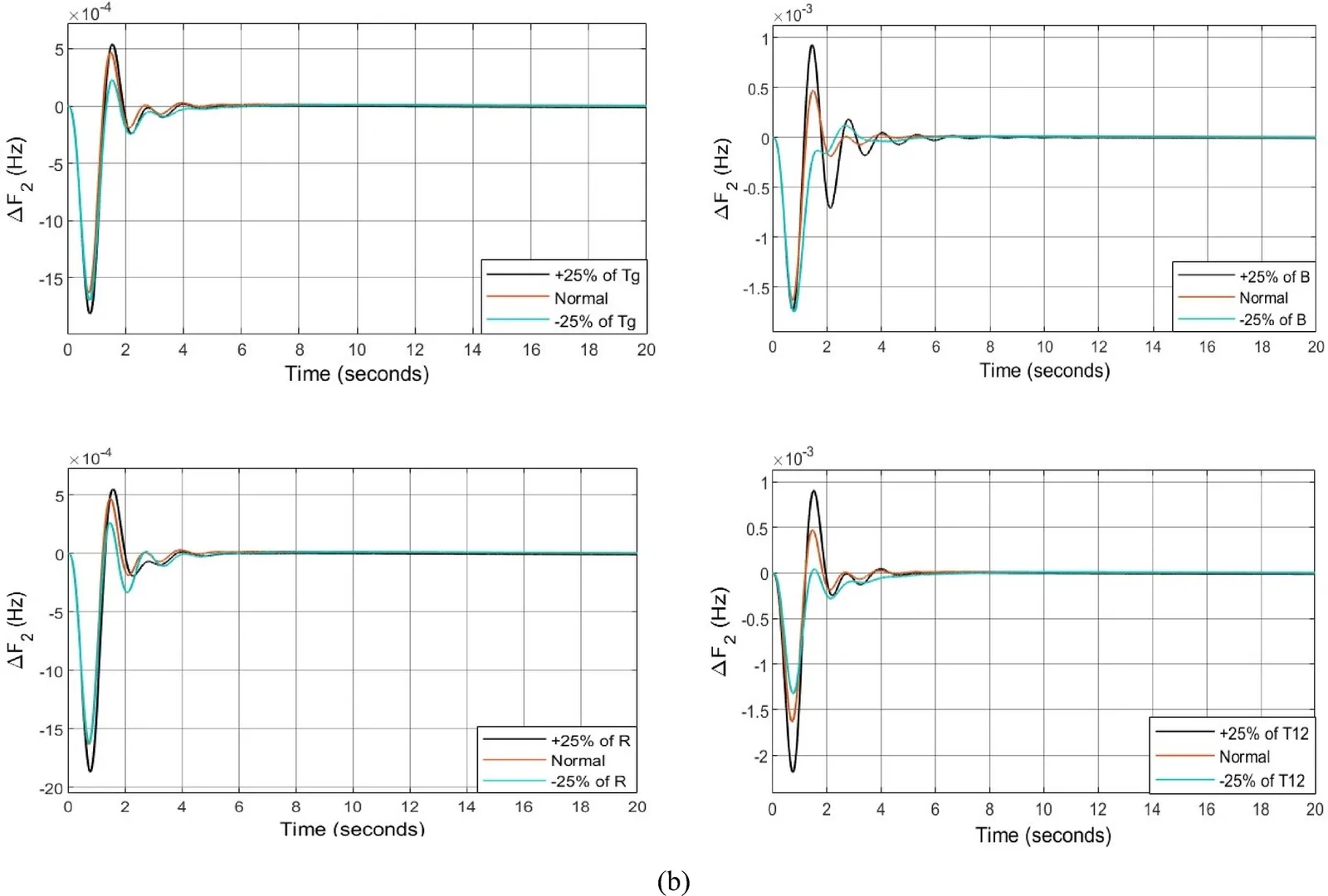

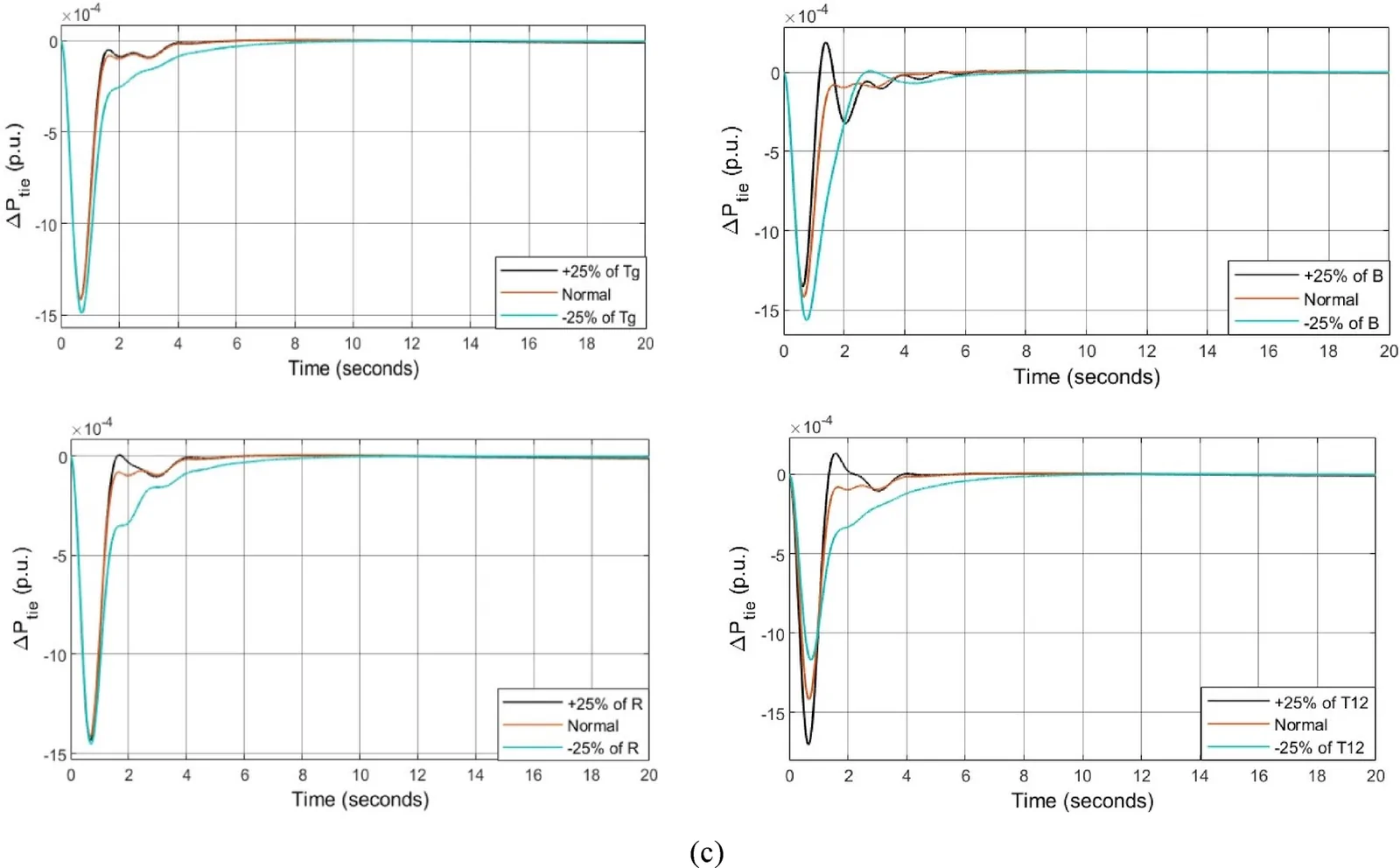
(**a**): Variation of ${T}_{g}$, *B*, *R*, and ${T}_{12}$ PS parameters for Δ ${F}_{1}$. (**b**): Variation of ${T}_{g}$, *B*, *R*, and ${T}_{12}$ PS parameters for Δ ${F}_{2}$. (**c**): Variation of ${T}_{g}$, *B*, *R*, and ${T}_{12}$ PS parameters for ΔP.

It is apparent from Fig. 12 that, when compared to other controllers in the existing literature, the WAA: (1 + FOPI)-FOPI-TID CC demonstrates consistent dynamic performance under randomized loading patterns in area 1. It shows reduced undershoot and overshoot, along with faster convergence, highlighting its robustness and effectiveness in handling dynamic variations Fig. 13.

**Case 4: sensitivity analysis/robustness**

**Fig. 14:**
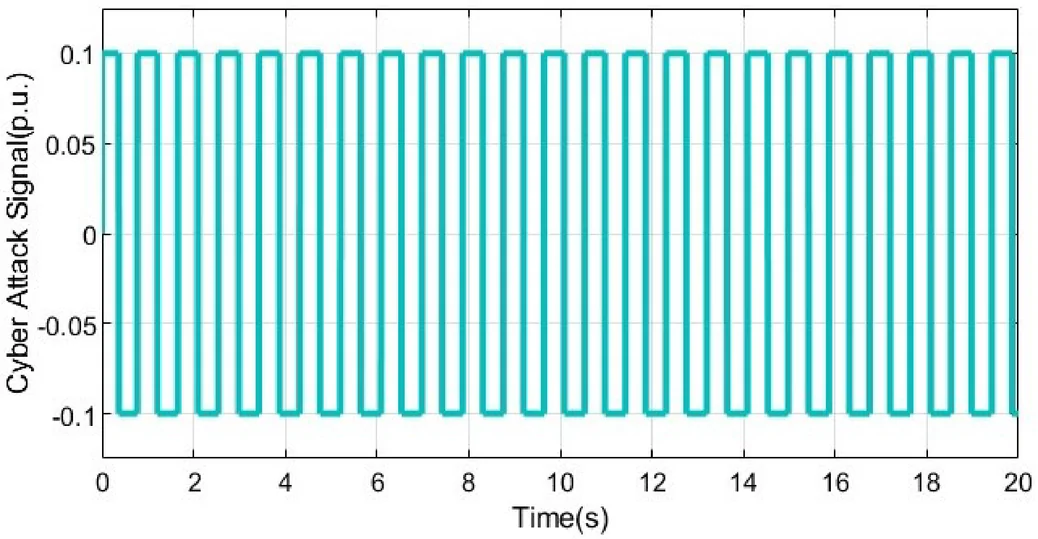
Cyber-attack signal model applied to the PS.

A sensitivity analysis was conducted to evaluate the robustness of the WAA optimized (1 + FOPI)-FOPI-TID CC. The stability of the proposed controller was assessed by comparing the responses of the original parameters to various metrics when parameters such as ${T}_{g}$*, **B**, **R**,* and ${T}_{12}$ are varied by approximately ± 25%, as outlined in Table 5 under $\Delta {P}_{L1}=1\%$ SLP at area 1. Table 5 and Fig. 14 demonstrate the suggested controller’s reliability under altered system settings. The impact of parameter fluctuations on the ITAE criterion, settling time, and undershoot achieved by the proposed approach under modifications of system parameters was found to be negligible. The analysis reveals that even with significant variations of up to 25% in parameters, the proposed scheme exhibits robustness and maintains excellent performance, with minimal effects on the key evaluation metrics. This underscores the robust nature of the controller and its ability to perform effectively under varying system conditions.

**Table 5:** Percentage variations in hybrid PS parameters.

Parameters	% of Change	ITAE	Settling time (0.0002 band) T_s_ (s)			Undershoot(-ve)		
			$\Delta {F}_{1} \Delta {F}_{2} \Delta P$			$\Delta {F}_{1}$ $\times {10}^{-3}$	$\Delta {F}_{2}$ $\times {10}^{-3}$	*Δ P* $\times {10}^{-4}$
${T}_{g}$	+ 25	0.01481	1.924	2.312	1.355	13.0	1.804	14.09
	Nominal	0.01381	1.693	1.752	1.355	13.0	1.625	14.13
	− 25	0.01691	2.169	2.333	2.376	13.0	1.687	14.87
*B*	+ 25	0.02249	3.883	2.432	2.333	13.0	1.713	13.49
	Nominal	0.01381	1.693	1.752	1.355	13.0	1.625	14.13
	− 25	0.02027	3.595	1.479	2.163	13.0	1.744	15.60
*R*	+ 25	0.01661	2.831	1.908	1.312	13.0	1.860	14.31
	Nominal	0.01381	1.693	1.752	1.355	13.0	1.625	14.13
	− 25	0.01736	2.788	2.333	2.461	13.0	1.612	14.50
${T}_{12}$	+ 25	0.01652	3.782	2.354	1.227	13.0	2.178	16.98
	Nominal	0.01381	1.693	1.752	1.355	13.0	1.625	14.13
	− 25	0.01962	3.970	2.454	3.014	13.0	1.320	11.70

**Case 5: Resonance Cyber Attack (ResA)**

In this case, the ResA is simulated by introducing load disturbances at arbitrary frequency values, without any prior knowledge of the system’s operational state. Figure 14. illustrates a cyber-attack injection signal applied to the power system model, represented as a bounded high-frequency square-wave disturbance varying between ± 0.1 p.u. over the simulation duration. This signal is used to emulate false data injection or malicious control signal manipulation, periodically perturbing system measurements or control inputs to evaluate the robustness of the load frequency control scheme under cyber-physical threats.

The cyber-attack signal is synthetically generated within the simulation environment using a periodic switching (square-wave) function with fixed amplitude and duty cycle.

**Fig. 15:**
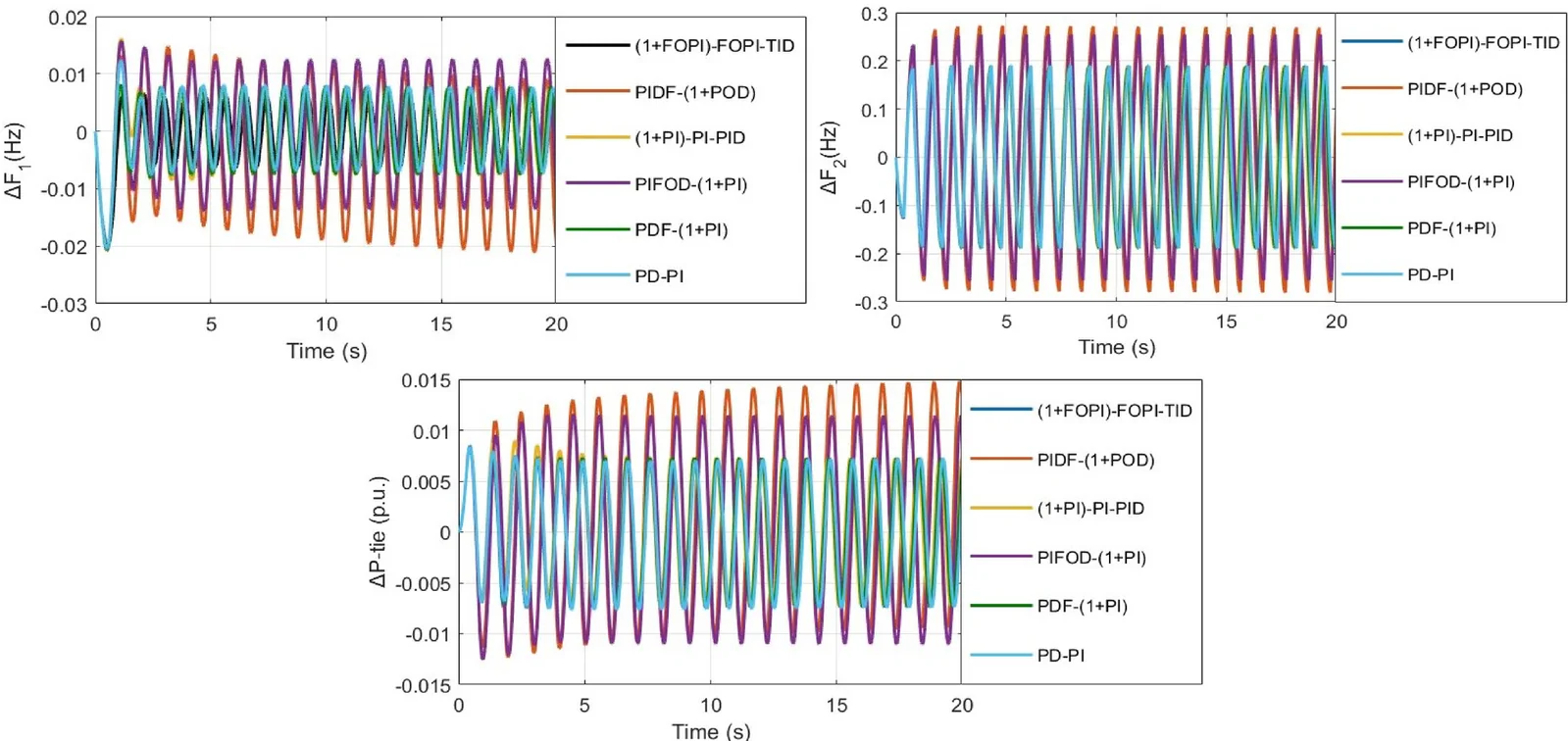
Controller performances under the cyber-attack: ΔF_1_, ΔF_2_, and ΔP_tie._

The dynamic response of the system to this attack is analyzed using various control strategies, with corresponding results illustrated in Fig. 15. Among the compared controllers, the WAA: (1 + FOPI)-FOPI-TID configuration demonstrates the most robust performance, exhibiting the lowest RoCoF amplitude in area 1 (0.0654) and maintaining a relatively moderate amplitude in area 2 (1.3276) summarized in Table 6. This indicates superior disturbance rejection and frequency stability under cyber-attack conditions.

**Table 6:** RoCoF (Hz/s) for different controllers.

Controller	Max. Amplitude (Area 1)	Max. Amplitude (Area 2)
WAA: (1 + FOPI)-FOPI-TID	**0.0654**	**1.3276**
WAA: PD-PI	0.0967	1.3835
WAA: (1 + PI)-PI-PID	0.1158	1.7025
WAA: PIFOD-(1 + PI)	0.0930	1.5391
WAA: PDF-(1 + PI)	0.0880	1.7652
PIDF(1 + FOD)	0.0959	1.5442

Significant values are in [bold].

The WAA: PDF-(1 + PI) controller ranks second, with a slightly higher RoCoF amplitude in area 1 (0.0880) and a comparable value in area 2 (1.7652), suggesting effective damping characteristics. In contrast, the (1 + PI)-PI-PID controller performs the poorest, recording the highest RoCoF amplitude in area 2 (1.7025), alongside a relatively elevated value in area 1 (0.1158). Similarly, the WAA: PIFOD-(1 + PI) controller shows inadequate performance in area 2 with an amplitude of 1.5391, further emphasizing its reduced resilience to cyber-attacks.

The RoCoF profiles, presented in Fig. 1, align with these observations, while most controllers effectively limit RoCoF within acceptable thresholds in area 1 and area 2 exhibits higher susceptibility to instability.

## Conclusion

**Fig. 16:**
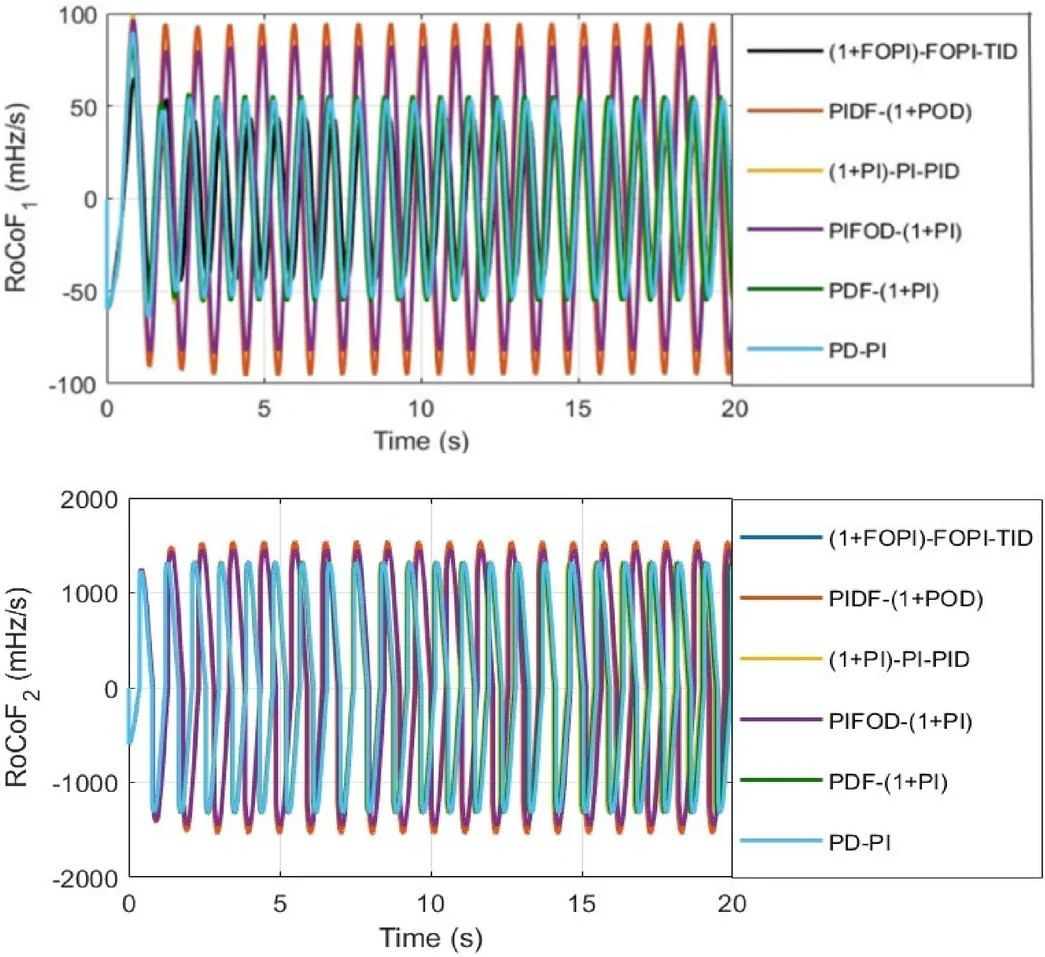
RoCoF results of PS areas for cyber-attack for Area-1 and Area-2.

This study presents a novel (1 + FOPI)-FOPI-TID cascade control strategy, optimized using a WAA, for the frequency regulation of heterogeneous two-area PSIs incorporating reheat thermal, gas, hydro, and nuclear generation units. The system model integrates critical nonlinearities, including GRC, BD, GDB, and CTD, as well as cyber-attack scenarios to evaluate real-world robustness. Extensive dynamic simulations, including random step load perturbations, are conducted to assess the effectiveness of the proposed control scheme. Comparative evaluations with contemporary control configurations, such as WAA: PIFOD-(1 + PI) and WAA: PD-PI CCs, demonstrate the superior dynamic performance of the proposed method. Specifically, the (1 + FOPI)-FOPI-TID controller achieved a 45.3% improvement in ITAE and reduced frequency settling times by 47.7% ($\Delta {F}_{1}$) and 32.8% ($\Delta {F}_{2}$) in the first and second areas, respectively, under test case 2. Moreover, sensitivity analysis confirms the robustness of the proposed controller, maintaining consistent performance despite substantial parameter variations. Under cyber-attack scenarios, the controller exhibited minimal RoCoF amplitudes in both areas, further underscoring its resilience Fig. 16.

In summary, while the fractional-order cascade structure is more complex than classical controllers, its fixed-parameter implementation post-tuning is feasible for real-time application, and the design-time optimization cost is justified by the achieved performance and robustness benefits. This research contributes significantly to the advancement of load frequency control strategies through the development of robust, WAA-based CC architecture. The WAA: (1 + FOPI)-FOPI-TID approach not only enhances system stability and dynamic responsiveness but also ensures operational resilience under nonlinearities and cybersecurity threats.

Future work will focus on implementing the controller on real-time hardware (like OPAL-RT) to empirically validate its computational performance, alongside the integration of fault-detection mechanisms capable of rapidly identifying and diagnosing malicious intrusions before they can destabilize the power system.

## Data Availability

The datasets used and/or analysed during the current study available from the corresponding author on reasonable request.

## Appendix

**Multi-unit multi area PS parameters** ^1,56,57^

$${B}_{1}={B}_{2}=0.4312\text{ p}.\mathrm{u}\frac{\mathrm{MW}}{\mathrm{Hz}}$$; $${R}_{1}={R}_{2}={R}_{3}=2.4 p.u.\frac{MW}{Hz}$$; $${T}_{sg1}={T}_{sg2}=0.08\text{ s}$$; $${T}_{t1}={T}_{t2}=0.3 s$$; $${K}_{r1}={K}_{r2}=0.3;$$ $${T}_{r1}={T}_{r2}=10 s;$$ $${K}_{PS1}={K}_{PS2}=68.9566 Hz /p.u.$$; $${T}_{12}=0.0433\text{ p}.\mathrm{u}.; {a}_{12}=-1;$$ $${T}_{w1}={T}_{w2}=1\text{ s};$$ $${T}_{RS1}={T}_{RS2}=5\text{ s}$$; $${T}_{RH1}={T}_{RH2}=78.75\text{ s}$$; $${T}_{GH1}={T}_{GH2}=0.2\text{ s}$$; $${X}_{C}=0.6 s$$; $${Y}_{C}=1\text{ s};$$ $${c}_{g}=1 s;$$$${b}_{g}=0.05 s;$$ $${T}_{F1}={T}_{F2}=0.23\text{ s}$$; $${K}_{T}=0.543478$$; $${K}_{H}=0.326084$$; $${K}_{G}=0.130438; {K}_{DC}=1; {T}_{DC}=0.2s;{T}_{CR1}={T}_{CR2}=0.01\text{ s}$$; $${T}_{CD1}={T}_{CD2}=0.2\text{ s}$$; $${T}_{PS1}={T}_{PS2}=11.49\text{ s}$$. **MSTA** **gains:** $${K}_{T}=0.543478,{K}_{H}=0.326084,\text{and }{K}_{G}=0.130438$$ ^1^.

**Nuclear** **power** **plant** **gains:** ${T}_{N}=0.05,{K}_{RN}=0.2, {T}_{N2}=0.25, {T}_{RHN}=10s, {T}_{RHN2}=10s, {T}_{RHN3}=0.25, {T}_{N3}=0.05s, \mathrm{and} {K}_{N}=0.25$ ^11^

## Abbreviations

LFC: Load frequency control
AGC: Automatic generation control
FO: Fractional order
RLP: Random step load
ITAE: Integral time multiplied absolute error
NRT: Non-reheat thermal
MSTA: Multi-source two-area
HVDC: High voltage direct current
*GDB*: Governor dead band
GRC: Generation rate constraints
CTD: Communication time delay
SLP: Sudden load perturbation
PID: Proportional-Integral-Derivative
TID: Tilt integral derivative
BD: Boiler dynamic
CC: Cascade controller
PSI: Power system interconnection
WWA: Weighted average algorithm
*i*: Related to number 1, 2, …, n
${P}_{L}$: Operating load (MW)
$\Delta {F}_{i}$: Frequency deviation (Hz)
$\Delta {P}_{Li}$: Step load change
$\Delta {P}_{tie}$: Power deviation in Tie-line (p.u.)
$${T}_{S}$$: Settling time (s)
*f*: Operating frequency (Hz)
${P}_{R}$: Rated Power for each area (MW)
${B}_{i}$: Bias frequency (p.u.MW/Hz)
${K}_{H}$: Contributing factors for hydro unit
${K}_{G}$: Contributing factors for gas unit
${K}_{DC}$: HVDC gain (Hz/p.u.)
${R}_{i}$: Regulation speed (Hz/p.u.)
$${T}_{ri}$$: Reheat TS (s)
${K}_{Ri}$: Reheat coefficient of steam turbine
${K}_{PSi}$: Power system gain (Hz/p.u.)
${T}_{ti}$: Time constant of steam turbine (s)
${T}_{gi}$: Time constant of speed governor (s)
$${T}_{12}$$: Synchronizing coefficient (p.u.)
${T}_{RSi}$: Reset time of hydro turbine (s)
${T}_{GHi}$: Time constant of hydro turbine (s)
${T}_{Wi}$: Nominal initial time of water in penstock (s)
${T}_{RHi}$: Hydro turbine speed governor droop time constant (s)
${T}_{Psi}$: Time constant of power system (s)
${K}_{T}$: Contributing factors for thermal unit
${T}_{DC}$: HVDC time constant (s)
${b}_{g}$: Gas turbine constant of positioner (s)
*c*_*g*_: Gas turbine valve positioner(s)
*Y*_*C*_: Lag time of gas turbine (s)
*X*_*C*_: Lead time of gas turbine (s)
${T}_{CRi}$: Time delay of Gas turbine combustion (s)
${T}_{CDi}$: Gas turbine compressor discharge volume (s)
${T}_{fi}$: Gas turbine fuel time constant (s)

## References

[1] Barakat, M. Optimal design of fuzzy-PID controller for automatic generation control of multi-source interconnected power system. *Neural Comput. Appl.* **34**, 18859–18880. 10.1007/s00521-022-07470-4 (2022).

[2] Barisal, A. K. Comparative performance analysis of teaching learning based optimization for automatic load frequency control of multi-source power systems. *Int. J. Electr. Power Energy Syst.* **66**, 67–77. 10.1016/j.ijepes.2014.10.019 (2015).

[3] Singh, K., Amir, M. & Arya, Y. Optimal dynamic frequency regulation of renewable energy based hybrid power system utilizing a novel TDF-TIDF controller. *Energy Sources Part. Recover Util. Environ. Eff.* **44** (4), 10733–10754 (2022).

[4] Daraz, A., Malik, S. A., Basit, A., Aslam, S. & Zhang, G. Modified FOPID controller for frequency regulation of a hybrid interconnected system of conventional and renewable energy sources. *Fractal Fract.* **7** (1). 10.3390/fractalfract7010089 (2023).

[5] Ali, T. et al. Terminal voltage and load frequency control in a real four-area multi-source interconnected power system with nonlinearities via OOBO algorithm. *IEEE Access*, (2024).

[6] Daraz, A. et al. Frequency regulation of interconnected hybrid power system with assimilation of electrical vehicles. *Heliyon* **10** (6), e28073. 10.1016/j.heliyon.2024.e28073 (2024). 38524527 PMC10958435

[7] Sargolzaei, A., Yen, K. K. & Abdelghani, M. N. Time-delay switch attack on load frequency control in smart grid. *Adv. Commun. Technol.* **5**, 55–64 (2013).

[8] Liu, S., Liu, X. P. & Saddik, A. E. Denial-of-Service (dos) attacks on load frequency control in smart grids, in *2013 ieee pes innovative smart grid technologies conference (isgt)*, 1–6. (2013).

[9] Wu, Y., Wei, Z., Weng, J., Li, X. & Deng, R. H. Resonance attacks on load frequency control of smart grids. *IEEE Trans. Smart Grid*. **9** (5), 4490–4502. 10.1109/TSG.2017.2661307 (2018).

[10] Panda, S., Mohanty, B. & Hota, P. K. Hybrid BFOA-PSO algorithm for automatic generation control of linear and nonlinear interconnected power systems. *Appl. Soft Comput. J.* **13** (12), 4718–4730. 10.1016/j.asoc.2013.07.021 (2013).

[11] Ye, Y., Daraz, A., Basit, A., Khan, I. A. & Alqahtani, S. A. Cascaded Fractional-Order Controller-Based Load Frequency Regulation for Diverse Multigeneration Sources Incorporated with Nuclear Power Plant, *Int. J. Energy Res.*, 10.1155/2024/7939416 (2024).

[12] Ogar, V. N., Hussain, S. & Gamage, K. A. A. Load frequency control using the particle swarm optimisation algorithm and PID controller for effective monitoring of transmission line. *Energies* **16** (15). 10.3390/en16155748 (2023).

[13] Chandran, K. et al. Modified cascade controller design for unstable processes with large dead time. *IEEE Access.* **8**, 157022–157036. 10.1109/ACCESS.2020.3019027 (2020).

[14] Pushkarna, M., Ashfaq, H., Singh, R. & Kumar, R. An optimal placement and sizing of type-IV DG with reactive power support using UPQC in an unbalanced distribution system using particle swarm optimization. *Energy Syst.* **15** (1), 353–370 (2024).

[15] Kumar, R., Singh, R., Ashfaq, H. & Kumar, S. Power system stability enhancement by damping and control of Sub-synchronous torsional oscillations using Whale optimization algorithm based Type-2 wind turbines. *ISA Trans. No Xxxx*. 10.1016/j.isatra.2020.08.037 (2020). 32888728

[16] Singh, K. & Arya, Y. Jaya-ITDF control strategy-based frequency regulation of multi-microgrid utilizing energy stored in high-voltage direct current-link capacitors. *Soft Comput. Fusion Found. Methodol. & Appl*, **27**, 9, (2023).

[17] Mohanty, B., Panda, S. & Hota, P. K. Controller parameters tuning of differential evolution algorithm and its application to load frequency control of multi-source power system. *Int. J. Electr. Power Energy Syst.* **54**, 77–85. 10.1016/j.ijepes.2013.06.029 (2014).

[18] Kumar, R., Diwania, S., Khetrapal, P., Singh, S. & Badoni, M. Sustainable computing: informatics and systems multimachine stability enhancement with hybrid PSO-BFOA based. *Sustain. Comput. Inf. Syst.* **32**, 100615. 10.1016/j.suscom.2021.100615 (2021).

[19] Kumar, R., Diwania, S., Khetrapal, P. & Singh, S. Performance assessment of the two metaheuristic techniques and their hybrid for power system stability enhancement with PV- STATCOM. *Neural Comput. Appl.* **34** (5), 3723–3744. 10.1007/s00521-021-06637-9 (2022).

[20] Sahoo, B. P., Panda, S., Padhy, S. & Panda, S. Simplified grey Wolf optimisation algorithm tuned adaptive fuzzy PID controller for frequency regulation of interconnected power systems. *Sustain. Energy Grids Networks*. **43** (1), 278–299. 10.1016/j.segan.2018.09.006 (2018).

[21] Altbawi, S. M. A. et al. Optimal design of fractional order PID controller based automatic voltage regulator system using gradient-based optimization algorithm. *J. King Saud Univ. - Eng. Sci.* **36** (1), 32–44. 10.1016/j.jksues.2021.07.009 (2024).

[22] Shalaby, R., El-Hossainy, M., Abo-Zalam, B. & Mahmoud, T. A. Optimal fractional-order PID controller based on fractional-order actor-critic algorithm. *3 Springer Lond.* **35** 10.1007/s00521-022-07710-7 (2023).

[23] Latif, A., Das, D. C., Barik, A. K. & Ranjan, S. Illustration of demand response supported co-ordinated system performance evaluation of YSGA optimized dual stage PIFOD-(1 + PI) controller employed with wind-tidal-biodiesel based independent two-area interconnected microgrid system. *IET Renew. Power Gener*. **14** (6), 1074–1086. 10.1049/iet-rpg.2019.0940 (2020).

[24] Barakat, M. H., Salama, G., Donkol, A. & Hamed, H. Optimal design of Fraction-Order Proportional-Derivative Proportional-Integral controller for Lfc of Thermal-Thermal-Wind turbines considering nonlinearities. *J. Adv. Eng. Trends*. **41** (2), 275–283. 10.21608/jaet.2021.64407.1090 (2021).

[25] Iqbal, M. S. et al. A hybrid optimization algorithm for improving load frequency control in interconnected power systems. *Expert Syst. Appl.* **249**, 103624. 10.1016/j.eswa.2024.123702 (2024).

[26] Shahi, M. N. S., Orka, N. A. & Ahmed, A. 2DOF-PID-TD: A new hybrid control approach of load frequency control in an interconnected thermal-hydro power system. *Heliyon* **10** (17), e36753. 10.1016/j.heliyon.2024.e36753 (2024). 39281473 PMC11401120

[27] Aryan, P. & Raja, G. L. Design and analysis of novel QOEO optimized parallel fuzzy FOPI-PIDN controller for restructured AGC with HVDC and PEV. *Iran. J. Sci. Technol. - Trans. Electr. Eng.* **46** (2), 565–587. 10.1007/s40998-022-00484-7 (2022).

[28] Arya, Y. AGC of PV-thermal and hydro-thermal power systems using CES and a new multi-stage FPIDF-(1 + PI) controller. *Renew. Energy*. **134**, 796–806. 10.1016/j.renene.2018.11.071 (2019).

[29] Priyadarshani, S., Subhashini, K. R. & Satapathy, J. K. Pathfinder algorithm optimized fractional order tilt-integral-derivative (FOTID) controller for automatic generation control of multi-source power system. *Microsyst. Technol.* **27** (1), 23–35. 10.1007/s00542-020-04897-4 (2021).

[30] Cheng, J. & De Waele, W. Weighted average algorithm: A novel meta-heuristic optimization algorithm based on the weighted average position concept. *Knowledge-Based Syst.* **305**, 112564 (2024).

[31] Guha, D., Roy, P. K. & Banerjee, S. Maiden application of SSA-optimised CC-TID controller for load frequency control of power systems. *IET Gener Transm Distrib.* **13** (7), 1110–1120. 10.1049/iet-gtd.2018.6100 (2019).

[32] Sahu, B. K., Pati, S., Mohanty, P. K. & Panda, S. Teaching-learning based optimization algorithm based fuzzy-PID controller for automatic generation control of multi-area power system. *Appl. Soft Comput. J.* **27**, 240–249. 10.1016/j.asoc.2014.11.027 (2015).

[33] Zheng, Y. et al. Load frequency active disturbance rejection control for multi-source power system based on soft actor-critic. *Energies* **14** (16), 1–17. 10.3390/en14164804 (2021).

[34] Khan, I. A. et al. November., Load frequency control in power systems with high renewable energy penetration: A strategy employing PIλ(1 + PDF) controller, hybrid energy storage, and IPFC-FACTS, *Alexandria Eng. J.*,** 106**, 337–366. 10.1016/j.aej.2024.06.087 (2024).

[35] Sahu, R. K., Panda, S. & Chandra Sekhar, G. T. A novel hybrid PSO-PS optimized fuzzy PI controller for AGC in multi area interconnected power systems. *Int. J. Electr. Power Energy Syst.* **64**, 880–893. 10.1016/j.ijepes.2014.08.021 (2015).

[36] Barakat, M. Novel chaos game optimization tuned-fractional-order PID fractional-order PI controller for load–frequency control of interconnected power systems. *Prot. Control Mod. Power Syst.* **7** (1). 10.1186/s41601-022-00238-x (2022).

[37] Ali, T., Malik, S. A., Daraz, A., Aslam, S. & Alkhalifah, T. Dandelion Optimizer-Based combined automatic voltage regulation and load frequency control in a Multi-Area, Multi-Source interconnected power system with nonlinearities. *Energies* **15** (22). 10.3390/en15228499 (2022).

[38] Zhang, P. et al. Multi-resolution based PID controller for frequency regulation of a hybrid power system with multiple interconnected systems, *Front. Energy Res.* **10**, 1–16, 10.3389/fenrg.2022.1109063 (2023).

[39] Daraz, A. et al. Automatic generation control of multi-source interconnected power system using FOI-TD controller. *Energies* **14**, 1–17. 10.3390/en14185867 (2021). PMC7678963 33216787

[40] Daraz, A. et al. Fitness dependent Optimizer-Based automatic generation control of Multi-Source interconnected power system with Non-Linearities. *IEEE Access.* **8**, 100989–101003. 10.1109/ACCESS.2020.2998127 (2020). PMC7678963 33216787

[41] Kalyan, C. H. N. S. & Rao, G. S. Impact of communication time delays on combined LFC and AVR of a multi-area hybrid system with IPFC-RFBs coordinated control strategy. *Prot. Control Mod. Power Syst.* **6** (1). 10.1186/s41601-021-00185-z (2021).

[42] Naga Sai, C. H. et al. Seagull Optimization Algorithm–Based Fractional-Order Fuzzy Controller for LFC of Multi-Area Diverse Source System With Realistic Constraints, *Front. Energy Res.* **10**, 1–14, (2022). 10.3389/fenrg.2022.921426

[43] Cavdar, B., Sahin, E., Sesli, E., Akyazi, O. & Nuroglu, F. M. Cascaded fractional order automatic generation control of a PV-reheat thermal power system under a comprehensive nonlinearity effect and cyber-attack. *Electr. Eng.* **105** (6), 4339–4360. 10.1007/s00202-023-01943-y (2023).

[44] Latif, A., Hussain, S. M. S., Das, D. C. & Ustun, T. S. Double stage controller optimization for load frequency stabilization in hybrid wind-ocean wave energy based maritime microgrid system, *Appl. Energy*, **282**, 116171, (2021). 10.1016/j.apenergy.2020.116171

[45] Prakash, A., Kumar, K. & Parida, S. K. PIDF(1 + FOD) controller for load frequency control with Sssc and ac-dc tie-line in deregulated environment. *IET Gener Transm Distrib.* **14** (14), 2751–2762. 10.1049/iet-gtd.2019.1418 (2020).

[46] Nayak, P. C., Prusty, R. C. & Panda, S. Grasshopper optimization algorithm optimized multistage controller for automatic generation control of a power system with FACTS devices. *Prot. Control Mod. Power Syst.* **6** (1). 10.1186/s41601-021-00187-x (2021).

[47] Awal, M., Atim, M. R., Wanzala, J. N., Obungoloch, J. & Barakat, M. Fire Hawk optimizer adjusted PD-PI cascade controller for automatic generation control of IPS. *Electr. Eng.* 10.1007/s00202-025-03009-7 (2025).

[48] Choudhary, R., Rai, J. N. & Arya, Y. Cascade FOPI-FOPTID controller with energy storage devices for AGC performance advancement of electric power systems, *Sustain. Energy Technol. Assessments*, **53**, 102671, (2022). 10.1016/j.seta.2022.102671

[49] Mao, J., Liu, R., Wu, A., Wu, S. & He, J. An Improved Whale Optimization Algorithm Based PIDF-(1 + PI) Cascade Automatic Generation Control for Multi-Area Multi-Source Power System with Capacitive Energy Storage, *IEEE Access*, 11, 72418–72435, (2023). 10.1109/ACCESS.2023.3250558

[50] Barakat, M., Donkol, A., Hamed, H. F. A. & Salama, G. M. Harris Hawks-Based optimization algorithm for automatic LFC of the interconnected power system using PD-PI cascade control. *J. Electr. Eng. Technol.* **16** (4), 1845–1865. 10.1007/s42835-021-00729-1 (2021).

[51] Barakat, M., Mabrouk, A. M. & Donkol, A. Optimal design of a cascade controller for frequency stability of photovoltaic–reheat thermal power systems considering nonlinearities. *Opt. Quantum Electron.* **55** (4), 1–24. 10.1007/s11082-023-04583-5 (2023).

[52] Barakat, M., Donkol, A., Hamed, H. F. A. & Salama, G. M. Controller parameters tuning of water cycle algorithm and its application to load frequency control of multi-area power systems using TD-TI cascade control. *Evol. Syst.* **13** (1), 117–132. 10.1007/s12530-020-09363-0 (2022).

[53] Priyadarshani, S., Subhashini, K. R. & Satapathy, J. K. *Pathfinder Algorithm Optimized Fractional order tilt – integral – derivative (FOTID) Controller for Automatic Generation Control of multi – source Power System*, **27**, (1) Springer Berlin Heidelberg, 23–35. 10.1007/s00542-020-04897-4 (2021).

[54] Piotrowski, A. P., Napiorkowski, J. J. & Piotrowska, A. E. Population size in particle swarm optimization. *Swarm Evol. Comput.* **58**, 100718. 10.1016/j.swevo.2020.100718 (2020).

[55] Ojha, S. K. & Obaiah, M. C. Optimization of a Novel FOPIDN-(1 + PIDN) controller for Renewable Integrated Multi-Area Load Frequency Control System with Non-linearities, *IEEE Access*, **13**, 56736–56755, (2025). 10.1109/ACCESS.2025.3555846

[56] Çelik, E. Design of new fractional order PI–fractional order PD cascade controller through dragonfly search algorithm for advanced load frequency control of power systems. *Soft Comput.* **25** (2), 1193–1217. 10.1007/s00500-020-05215-w (2021).

[57] Gheisarnejad, M. An effective hybrid harmony search and cuckoo optimization algorithm based fuzzy PID controller for load frequency control. *Appl. Soft Comput. J.* **65**, 121–138. 10.1016/j.asoc.2018.01.007 (2018).

